# The lifetime prevalence and associated factors of suicidal ideation and suicide attempts among high school adolescents in Ethiopia: a systematic review and meta-analysis

**DOI:** 10.1186/s13034-025-00927-z

**Published:** 2025-06-04

**Authors:** Berhan Tekeba, Tadesse Tarik Tamir, Derese Abebe Gebrehana, Yohannes Abich, Nebebe Demis Baykemagn, Melaku Alelign Mengstie, Alebachew Ferede Zegeye

**Affiliations:** 1 Department of Pediatrics and Child Health Nursing, School of Nursing, College of Medicine and Health Sciences, University of Gondar, Gondar, Ethiopia; 2 Department of Medical Nursing, School of Nursing, College of Medicine and Health Sciences, University of Gondar, Gondar, Ethiopia; 3 Departemnt of Internal Medicine, School of Medicine, College of Medicine and Health Sciences, University of Gondar, Gondar, Ethiopia; 4 Department of physiotherapy, College of Medicine and Health sciences, University of Gondar, Gondar, Ethiopia; 5 Department of Health Informatics, Institute of Public Health, College of Medicine and Health Sciences, University of Gondar, Gondar, Ethiopia; 6 Department of Information science, College of informatics, University of Gondar, Gondar, Ethiopia

**Keywords:** Suicide, High school adolescents, Suicidal ideation, Suicidal attempt, Ethiopia

## Abstract

**Introduction:**

Suicide is a major public health problem and one of the top causes of death among adolescents worldwide. The Ethiopian government works to ensure healthy lives and promote well-being in adolescents through the National Adolescent and Youth Mental Health Strategy. Despite these efforts, suicide in adolescents remains pressing in Ethiopia; however, study findings regarding suicidal ideation and suicide attempts and their associated factors in high school adolescents have been inconsistent and non-conclusive. Therefore, this review aimed to assess the pooled national prevalence and risk factors of suicidal ideation and suicide attempts among high school adolescents in Ethiopia.

**Methods:**

The Preferred Reporting Items for Systematic Review and Meta-analysis (PRISMA) guideline was followed for this systematic review. We searched PubMed, Embase, ScienceDirect, and Google Scholar databases. We included all observational studies that report the prevalence and associated factors of suicidal ideation and suicide attempts among high school adolescents in Ethiopia. Using a standard data extraction format, two authors separately extracted all required data. The meta-analysis was conducted using Stata version 17 statistical software. The Cochrane Q test and I² statistics were employed to evaluate the heterogeneity among the included studies. Pooled prevalence estimates, along with their 95% confidence intervals, were calculated using a random-effects model. Potential sources of heterogeneity across studies were further explored through subgroup analyses, sensitivity analysis, and meta-regression analysis.

**Result:**

The review comprised ten studies with 8,620 participants. Out of 1,451 studies identified, 10 studies were included in the analysis. As the random effect model indicated that the pooled prevalence of suicide ideation and suicide attempt among high school adolescents in Ethiopia was 16% (95% CI: 12%, 19%) and 10% (95% CI: 6%, 13%), respectively. The highest prevalence of suicidal ideation was observed after the country’s implementation of HSTP II [18 (95% CI: 15–22), I²=83.94, P-value < 0.001], while the lowest prevalence was depicted in adolescents aged 10–19 years [11% (95% CI 10, 13), I²=93.3, P-value < 0.001]. Similarly, the highest pooled prevalence of suicide attempt was observed after the country’s implementation of HSTP II [12% (95% CI: 10–13), I²=67.77, P-value < 0.03], while the lowest prevalence was depicted in adolescents aged 10–19 years [5% (95% CI 1, 9), I²=94.43, P-value < 0.001]. The sensitivity analysis indicated that none of the point estimates were outside of the overall 95% confidence interval. No publication bias was seen in suicide ideation. But evidence of publication bias for suicide attempts was identified through the left trim and fill analysis. Gender, disappointment with school results, family history, alcohol use, the presence or absence of family or social support, history of abuse, living arrangement of adolescent, anxiety, and depression were significantly associated with suicide ideation and suicide attempts among high school adolescents in Ethiopia.

**Conclusion:**

This review revealed that a significant proportion of high school adolescents had suicidal ideation and suicide attempts in Ethiopia. Therefore, the government and all stakeholders should track the outcome of the suicide prevention project under HSTP II and other initiatives. In addition, emphasis should be given to disadvantaged adolescents, including females, orphans, alcohol users, and mentally challenged adolescents. Furthermore, school-based interventions like social support and suicide prevention initiatives shall be promoted.

## Introduction

Suicide is defined as deliberately trying to kill oneself. Suicidal ideation is any self-reported desire to harm oneself that is not accompanied by any preparatory behavior. A suicide attempt, on the other hand, is a non-fatal outcome that is instigated and perpetrated by the person in question and culminates in self-harm [1, 2]. Suicidal behavior is a public health concern globally over the life span of teenagers aged 14–18 years, with particular preventative challenges [3]. Suicide is the second most common cause of death worldwide for youths aged 10 to 24 [4]. Suicidal ideation (thinking) frequently precedes suicide attempts, with more than one-third of adolescents with ideation eventually attempting suicide [5].

Teenagers are at a higher risk of engaging in suicidal behavior because of the biological, cognitive, and psychosocial transitions that occur during this developmental stage, including a strong desire to try new behaviors, a low perception of risky behavior, and demands for independence [6]. In addition, academic strain, peer pressure, bullying, identity and self-esteem concerns, and familial and environmental stressors such as conflict, abuse, and neglect predispose teenagers to suicidal ideation. Furthermore, the adolescent brain, specifically the prefrontal cortex, is developing. This has an impact on emotional regulation, impulse control, and problem-solving, making it difficult for teenagers to deal with uncomfortable feelings or contemplate long-term repercussions [7–10].

The factors found to be associated with suicidal ideation and suicide attempts among adolescents in low- and middle-income countries were female gender [11], loneliness [12], sadness [13], anxiety [14], depression [15, 16], alcohol use [17], substance use [17], school absenteeism [18], poor social support [19], and experience of violence [20]. However, there remains a notable lack of up-to-date and comprehensive evidence on suicidal ideation and attempts, as well as their risk factors, among high school adolescents in Ethiopia. This gap is particularly relevant in the context of the country’s completion of the Health Sector Transformation Plan II, the resilience measures following the COVID-19 pandemic, and the increasing influence of globalization, which has amplified social media use and its detrimental effects, including cyber bullying.

The World Health Organization (WHO) works on strategies, programs, and tools, including Helping Adolescents Thrive (HAT), to assist governments in responding to the health needs of adolescents [21]. The Ethiopian government also works to achieve the Sustainable Development Goals (SDG-3) and HTSP II to ensure healthy lives and promote well-being in adolescents through the National Adolescent and Youth Mental Health Strategy (2021–2025). Despite these efforts, suicide in adolescents remains pressing in Ethiopia, where mental health services are underdeveloped and stigma around mental health issues persists [22, 23]. In Ethiopia, social stigma and a lack of accessible mental health services exacerbate suicide ideation and suicide attempts in adolescents [24, 25]. On the other hand, Ethiopia’s deeply rooted religious traditions, which view suicide as sin, can play a protective role in suicide prevention [26]. Moreover, in Ethiopia, most adolescents live with their parents, by which a supportive family environment, parental monitoring, and a sense of belonging may protect adolescents from suicidal thoughts [27]. However, suicide remains a significant concern among adolescents in Ethiopia.

Prior studies in Ethiopia couldn’t address the pooled burden and risk factor of the problem in these high-risk groups (adolescents). In addition, there is a variation in studies reporting the prevalence and risk factors of suicidal ideation and suicide attempts among high school adolescents in Ethiopia. For instance, one study found that as high as 22.5% and 16.2% [28] of high school adolescents had suicidal ideation and suicide attempts, respectively; on the other hand, one study estimated that as low as 5.85% [29] and 5.5% [30] of adolescents had suicidal ideation and suicide attempts, respectively. Therefore, to come up with a consistent and conclusive report on the prevalence and associated factors of suicidal ideation and suicide attempts among high school adolescents in Ethiopia, this review intended to assess the pooled national prevalence and risk factors of suicidal ideation and suicide attempts among high school adolescents in Ethiopia. Findings from this review will be useful in developing specific evidence-based prevention and treatment strategies. This review finding will also be used as input information for the pediatric suicide prevention implementation research package conducted in Ethiopia.

## Methods

### Study setting, design and period

A systematic review of published studies was used to determine the lifetime prevalence of suicidal ideation and suicide attempts among high school adolescents in Ethiopia. Ethiopia is situated in the Horn of Africa. It is bordered by Eritrea to the north, Djibouti and Somalia to the east, Sudan and South Sudan to the west, and Kenya to the south. Currently, the Ethiopian population is estimated to be 129,384,266, with 20% living in urban areas [9, 10]. The adolescents and youth in Ethiopia comprise 33% of the Ethiopian population [21]. As of a recent estimate, 4.27 million students were enrolled in secondary education [31]. As of the 2022/2023 national report, the total number of students in Ethiopia, excluding the Tigray region, from grades 9 to 12 was 2,829,719, consisting of 1,383,422 males and 1,446,272 females [21]. The specific age at which adolescents typically begin high school in Ethiopia is not fixed and exhibits considerable variation across different regions within the country. To ensure comprehensive inclusion and avoid overlooking individuals within this demographic, our analysis encompassed adolescents aged 10 to 24 years.

### Systematic review registration, data source and search strategy

The preferred reporting items for the Systemic Review and Meta-analysis (PRISMA) checklist were utilized to develop the systematic review methodology [32]. This systematic review and meta-analysis was registered in the International Prospective Register of Systematic Reviews (PROSPERO) with protocol number CRD42024618211.

To find potentially relevant articles, a thorough search of studies done from 2000 to 2023 was conducted in the following databases: PubMed, Embase, ScienceDirect, and Google Scholar. All searches were restricted to articles written in English. Studies identified by our search strategy were retrieved and managed with EndNote X7 software. The keywords for the context, condition, and population were combined using Boolean words, including OR and AND, for searching. The search strategy included medical subject headings (MeSH terms, Emtree). As a result, the core searching keywords used were “Prevalence AND “associated factor” AND “suicidal ideation” OR “suicide attempts” AND “high school adolescents” AND Ethiopia.” The search strategy used in different databases was presented in the table below (Table 1).

**Table 1:** Different search engines and results for systematic review and meta-analysis of the lifetime prevalence and associated factors of suicidal ideation and suicide attempts among high school adolescents in Ethiopia

Source	Search engine	# of research article
Google scholar	The prevalence and associated factors of suicidal ideation and suicide attempts among high school adolescents in Ethiopia Ethiopia “adolescent “	900
PubMed	(((((((((((((((((((((Prevalence[Title/Abstract]) OR (Magnitude[Title/Abstract])) OR (Burden[Title/Abstract])) OR (Epidemiology[Title/Abstract])) OR (Incidence[Title/Abstract])) OR (Proportion[Title/Abstract])) AND (“associated factor*“[Title/Abstract])) OR (“risk factor*“[Title/Abstract])) OR (“contributing factor*“[Title/Abstract])) OR (predictor*[Title/Abstract])) OR (determinant*[Title/Abstract])) AND (“suicidal ideation“[Title/Abstract])) OR (“suicide attempts“[Title/Abstract])) OR (“self-harm“[Title/Abstract])) OR (“self-injury“[Title/Abstract])) OR (suicide[Title/Abstract])) AND (adolescent*[Title/Abstract])) OR (youth*[Title/Abstract])) OR (teenager*[Title/Abstract])) OR (“high school adolescent*“[Title/Abstract])) OR (“preparatory school student*“[Title/Abstract])) AND (Ethiopia[Title/Abstract])	464
Embase	(‘prevalence’/exp OR ‘prevalence’ OR ‘magnitude’/exp OR ‘magnitude’ OR ‘burden’/exp OR ‘burden’ OR ‘incidence’/exp OR ‘incidence’) AND (‘associated factor’/exp OR ‘associated factor’ OR ‘risk factor’/exp OR ‘risk factor’ OR ‘predictors’/exp OR ‘predictors’ OR ‘determinants’/exp OR ‘determinants’) AND (‘suicidal ideation’/exp OR ‘suicidal ideation’ OR ‘suicide attempt’/exp OR ‘suicide attempt’) AND (‘high school student’/exp OR ‘high school student’ OR ‘adolescent’/exp OR ‘adolescent’ OR ‘young adult’/exp OR ‘young adult’) AND (‘ethiopia’/exp OR ‘ethiopia’)	39
Since direct	Prevalence AND “associated factor"AND “suicidal ideation” OR “suicide attempts” AND “high school adolescents” OR “secondary school students” AND Ethiopia	48

### Eligibility criteria

#### Inclusion criteria

The review utilized CoCoPop criteria to screen for inclusion and exclusion. The condition (Co) was the prevalence and risk factors of suicidal ideation and suicide attempts, the context (Co) was the only study in Ethiopia, and the study population (Pop) was high school students/adolescents. The study included all observational studies conducted in Ethiopia that reported the prevalence and associated factors of suicidal ideation and suicide attempts among high school students/adolescents with findings reported in the English language.

#### Exclusion criteria

Studies that were conducted in languages other than English, articles with incomplete information, and qualitative studies were excluded from the study.

#### Outcome of interest

The second outcome of this study was to identify the associated factors of suicidal ideation and suicide attempts among high school students in Ethiopia. We used the odds ratio calculated in Stata from the two-by-two table for determining the association between suicidal ideation and suicide attempts and its associated factors. The associated factors for this review included socio-demographic characteristics, family history, and psychological problems. The primary outcome was determined from 10 studies, and the second objective was determined from variables repeatedly done in more than two studies. Incomplete reporting for independent variables was excluded from the analysis.

### Data extraction

Data were extracted from articles included in the review using a standardized data extraction format adapted from the Joanna Briggs Institute (JBI) by our team with clear inclusion and exclusion criteria. Any disagreement during the data extraction was resolved through discussion and consensus (a Delphi process). The name of the first author and year, the study subject’s exposure to suicidal ideation and suicide attempt, and the sample size were used to determine the prevalence of suicidal ideation and suicide attempts. For the second objective (factors), data were extracted in a format of two-by-two tables, and then the log odds ratio for each factor was calculated based on the findings of the original studies. For different classifications of independent variables among different studies, re-categorization was done by cross-tabulation and calculating the new odds ratio and confidence interval.

### Risk of bias (quality) assessment

All articles selected for inclusion in the review were assessed rigorously by review authors (YA, MAM, and TTT). To assess the risk of bias within included studies, the Newcastle-Ottawa Scale (NOS) was used. The NWS scale was developed to assess the quality of an observational study with its design, content, and ease of use directed to the task of incorporating the quality [33]. The tool mainly includes (1) representativeness of the sample, (2) sample size, (3) non-respondents, (4) ascertainment of exposure or risk factors, and (5) comparability of subjects in different outcome groups. (6) Assessment of outcome; (8) statistical test. Each of the above scales has its items. The values of items range from 0 to 10. Studies that had 9–10 points were considered “very good.” Studies that get 7–8 points are considered “good” quality. Studies with 5–6 points are considered “satisfactory studies.” Studies with 0 to 4 points were considered “unsatisfactory” [33]. In this systematic review and meta-analysis, they were classified as having very good and good qualities based on their points of assessment. All the studies included in the systematic review and meta-analysis were ten [19, 28–30, 34–39].

### Data synthesis and analysis

The data entry and statistical analysis were carried out in Comprehensive Meta-analysis [40] using SATA version 17 software after extraction from Microsoft Excel format. Tables and figures were used to summarize the selected studies and results descriptively. We also implemented a meta-analysis of studies to provide a comparable classification of the determinants, exposure, and outcome variables. For the purpose of meta-analysis, we considered estimates of the adjusted odds ratio with a confidence interval (CI) as the measure of association. The overall effect (pooled estimate of the magnitude and factors of suicidal ideation and suicide attempt) was estimated using a random effect model and measured by the prevalence rate and odds ratio with a 95% CI. We selected the random effect model because of heterogeneity due to differences in the studies. To determine heterogeneity among studies, we calculated the I² statistic, which describes the percentage of total variation among studies due to heterogeneity rather than chance. An I^2^-static’s value of 30–60% may represent moderate heterogeneity, 50–90% may represent substantial heterogeneity, and 75–99% represents the highest heterogeneity [41, 42]. Furthermore, sensitivity analysis was conducted to assess the stability or robustness of the pooled estimate to outliers and the impacts of individual studies. Due to heterogeneity among studies, we performed subgroup analysis based on study subjects risk exposure, sample size, study setting, study quality, publication year, sample size, COVID-19 era, study subjects age, and country policy (HSTP II). In order to check publication bias, funnel plot asymmetry and the Egger test of intercept random effects models were used [43]. A random effects model was used to estimate the pooled estimate. Point prevalence, as well as 95% CI, was presented in the forest plot format. In this plot, the size of each box indicates the weight of the study, while each crossed line refers to the 95% CI. For the second outcome, a chi-square report was used to determine the association between determinant factors and suicidal ideation and suicide attempt.

## Result

### Study identification and characteristics of included studied

**Fig. 1:**
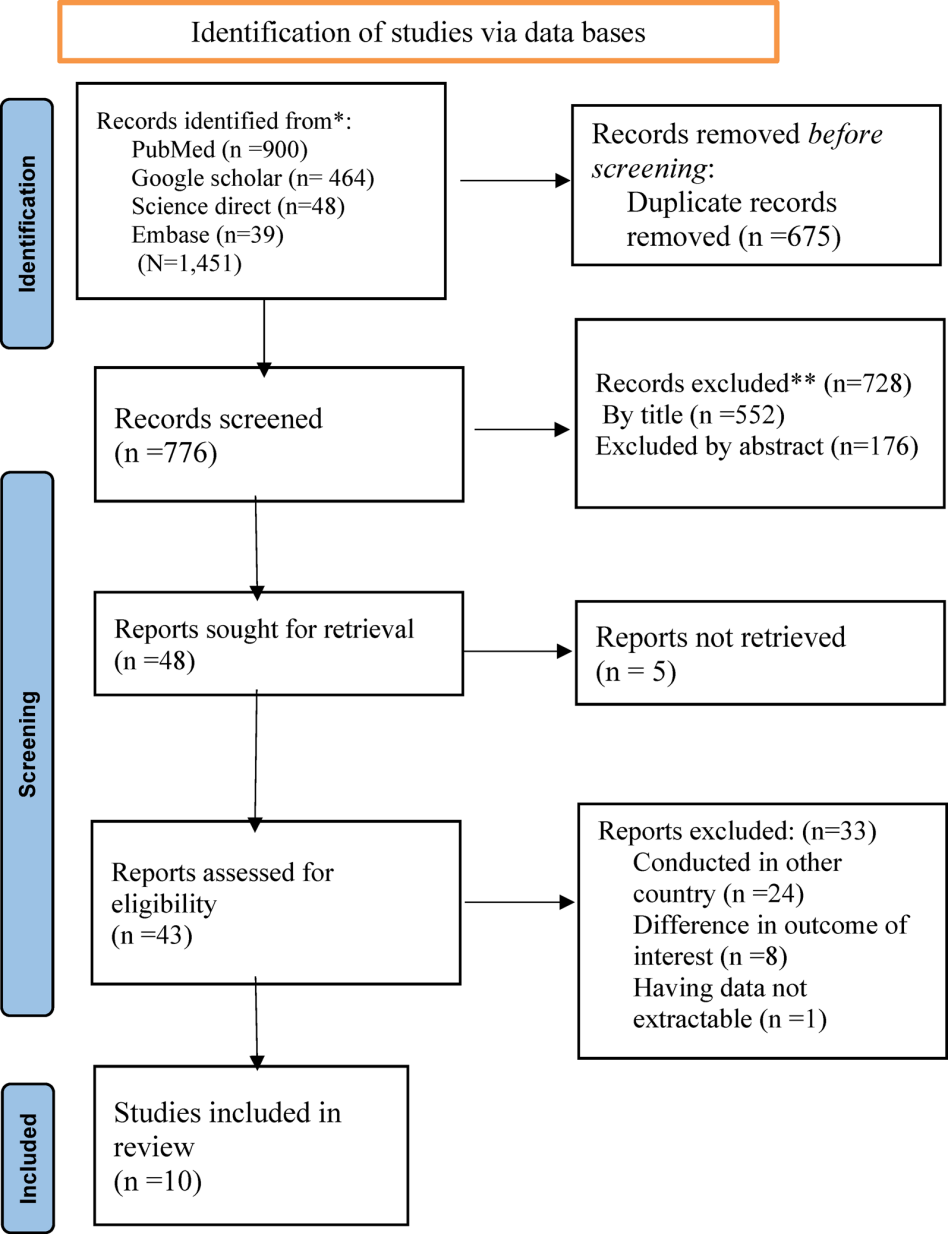
Flowchart diagram describing the selection of studies included in the systematic review and meta-analysis using the 2020 PRISMA checklist

A systematic search was conducted from May 20 to November 20, 2024, which yielded a total of 1,451 articles published between 2000 and 2024, as illustrated in Fig. 1. Of these, 675 articles were excluded due to duplication. An additional 552 articles were discarded following a review of their titles. Among the remaining 224 articles, 176 were deemed irrelevant based on their abstracts. Following a detailed evaluation of the full texts of 43 articles assessed for eligibility, only ten studies met the predefined inclusion criteria. Consequently, this systematic review and meta-analysis were conducted using these ten eligible studies.

 [44–46] (Fig. 1).

### Characteristics of the included studies

The characteristics of the items included are presented below (Table 2). A total of 8,620 adolescents from ten studies published from 2000 to 2024 were included in the systematic review and meta-analysis. Regarding sample size, the sample size of studies ranged from 372 to 2709 [37, 38]. Most of the studies (*n* = 6) were done in the Amhara region [19, 29, 30, 34, 37, 38]. Six (*n* = 6) studies [19, 28, 29, 34–36, 39] were done in pure urban areas, and four (*n* = 4) studies were done in mixed urban-rural areas [30, 35, 37, 38]. Eight studies (*n* = 8) were done at the zone level, and two (*n* = 2) studies were from the regional level [29, 37]. Three of the studies were done on study subjects with a risk/exposure to suicide, including a study done in war-affected areas (Amhara region) during the Tigray-Ethiopian conflict, studies with substance-using youths, and studies among female adolescents [19, 37, 38]. Table 1.

**Table 2:** Characteristics of study on prevalence and associated factor of suicidal ideation and suicide attempt among high school adolescents in Ethiopia

Author name (reference)	Publication year	Region	Data collection tool	SS	Associated factors	prevalence	
						Suicidal ideation	Suicidal attempt
Aliy et al. [28]	2023	Oromia	WMH-CIDI	1,114	Sex, age, inattention, school result, alcohol use, cyber bullying, family Hx of ideation/attempt, comorbidities, family environment, anxiety, depression	22.5	13.3
Amare et al. [34]	2015	Amhara	WHO-CIDI	573	Disappoint with grade result, living arrangement, school absence, loneliness, hopelessness, social support, physically hearted, drunk alcohol	22.5	16.2
Bete et al. [35]	2023	Harrer	WMH- CIDI	1,666	Gender, marital status, social support, depression, anxiety, stress, family Hx of mental illness, sleep quality, current chat user, ever sexually abused, gender, residence	13.82	7.61
Germew et al. [30]	2023	Amhara	WHO-self report questionary	1,597	Age, occupation, marital status, income, mental health problem, violence, substance abuse, risky sexual practice,	9.9	5.5
Giru et al. [36]	2012	Oromia	WHO-CIDI	758	Sex, grade, ethnicity, social support, disappointed with school result, family Hx of suicide, loneliness, hopelessness, worry, alcohol, chat	20.5	12.5
Jessica et al. [37]	2014	Amhara		2,709	Marital status, age, always lived in community, parent supervision, parent education, employed, current school attendance, household wealth, residence, depression, community involvement	11.23	2.28
Kassa et al. [19]	2023	Amhara	WHO-CIDI	668	Sex, residency, PTSD, chronic medical illness, family history of suicide, social support, ever alcohol drinking, current alcohol drinking, depression, anxiety	16.29	12.18
Melkam et al. [38]	2022	Amhara	ASIST, DASS-21	372	Sex, age, alcohol use, friend substance use, social support, stress, anxiety, depression, loss of beloved one	14.5	9.9
Molla et al. [39]	2019	SNNPR	WHO-CIDI	517	Sex, living status, stress, current time use of khat, social support	12.4	7.2
Shifraw et al. [29]	2006	Amhara		667	Age, sex, living arrangement	5.85	6.6

### Pooled prevalence of suicidal ideation and suicide attempt among high school adolescents in Ethiopia

**Fig. 2:**
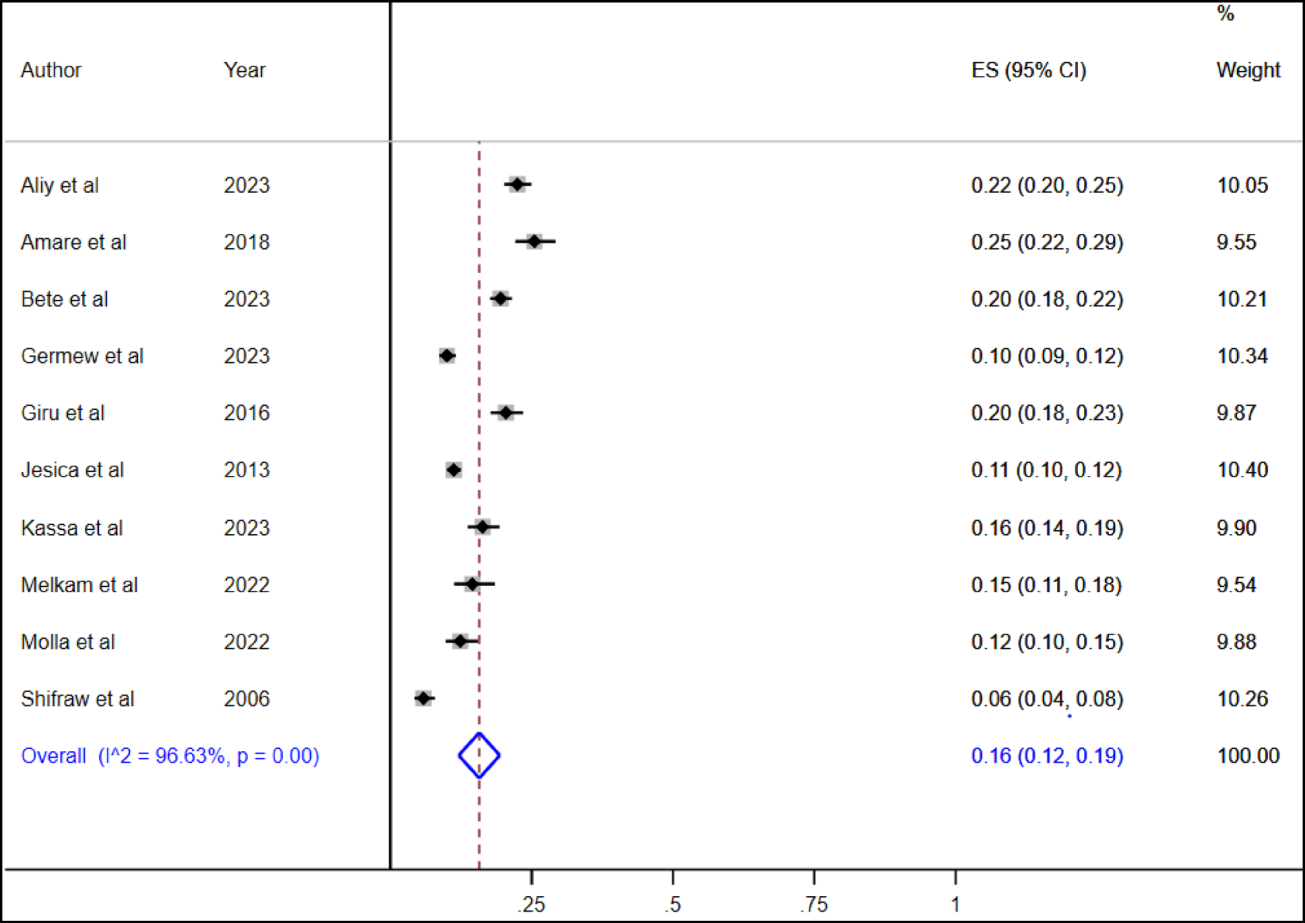
The overall pooled life time prevalence of suicidal ideation among high school adolescents in Ethiopia

**Fig. 3:**
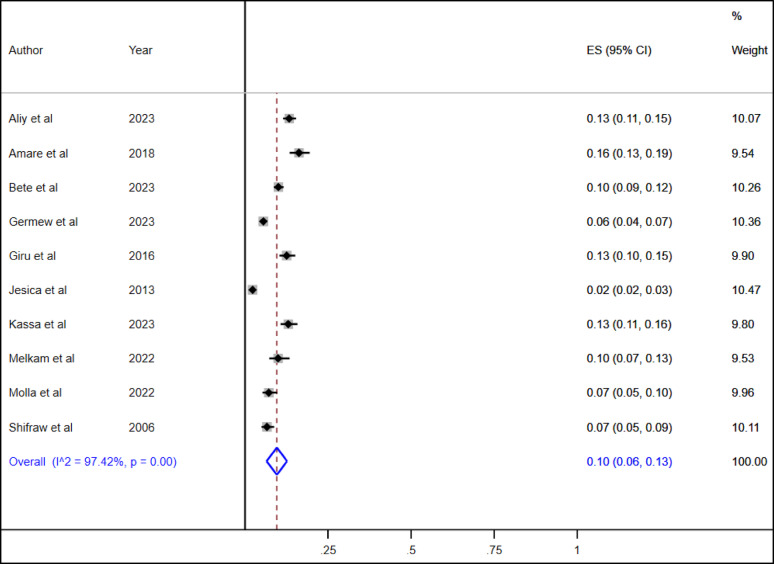
the overall pooled life time prevalence of suicidal attempt among high school adolescents in Ethiopia

A total of ten studies were included in this systematic review and meta-analysis to estimate the pooled prevalence of suicidal ideation and suicide attempts among high school adolescents in Ethiopia. The overall pooled lifetime prevalence of suicidal ideation and suicide attempt among high school adolescents was 16% (95% CI: 12, 19) and 10% (95% CI: 6, 13), respectively (Figs. 2 and 3).

### Heterogeneity tests

Since significant heterogeneity was observed from the random effect pooled estimate, we made a subgroup analysis, sensitivity analysis, DOI plot analysis, trim and fill analysis, and meta-analysis to handle heterogeneity. However, the source of heterogeneity was not identified.

### Sub group analysis

Due to significant heterogeneity between studies observed in our analysis (I² = 96.63 for suicidal ideation and I² = 97.42 for suicidal attempt, with both significant p-values < 0.001), subgroup analysis has been done on study subjects’ risk exposure, sample size, study setting, study quality, publication year, sample size, COVID-19 era, study subjects’ age, and country policy (HSTP II). The highest prevalence was observed after the country’s implementation of HSTP II [18% (95% CI: 15–22), I²=83.94, P-value < 0.001], while the lowest prevalence was depicted in adolescents aged 10–19 years [11% (95% CI 10, 13), I²=93.3, P-value < 0.001]. Similarly, the highest pooled prevalence of suicidal attempts was observed after the country’s implementation of HSTP II [12% (95% CI: 10–13), I²=67.77, P-value < 0.03], while the lowest prevalence was depicted in adolescents aged 10–19 years [5% (95% CI 1–9), I²=94.43, P-value < 0.001]. Subgroup analyses were also conducted among youths (study subjects) at risk of suicide, including those from war-affected areas in the Tigray-Ethiopian conflict, substance-using youths, female adolescents, and those without these risk factors. Accordingly, the lowest prevalence of suicidal ideation and suicide attempt was observed in risky study subjects [14% (95% CI 10–17), I²=97.9, p-value < 0.001] and [8% (95% CI 1–16), I²=97.6, p-value < 0.001], respectively. The higher prevalence was observed in studies without the above risk exposure, which was [17% (95% CI: 11–12), I² = 97.57, p-value < 0.001] for suicidal ideation and [10% (95% CI: 7–13), I² = 93.75, p-value < 0.001] for suicide attempt.

Discrepancy in prevalence of suicidal ideation and suicide attempt was observed in different parameters of studies as described in Table 3 below. While subgroup analysis was conducted, the heterogeneity persisted in many subgroup analyses, suggesting the presence of additional influencing factors.

**Table 3:** sub-group analysis of suicidal ideation and suicide attempt among high school adolescents in Ethiopia

			Suicidal ideation		Suicidal attempt	
Sub-group	Variable	Number of studies	Prevalence (95%, CI)	Heterogeneity (I^2^), *p*-value	Prevalence (95%, CI)	Heterogeneity (I^2^), *p*-value
Study subjects	Risky	3	14 (10,17)	97.9, < 0.001	8 (1,16)	97.6, < 0.001
	Non-risky	7	17 (11,22)	97.57, < 0.001	10 (7,13)	93.75, < 0.001
Study setting	Urban	6	17 (11,24)	97.35, < 0.001	11 (8,14)	90.57, < 0.001
	Semi-urban	4	14 (10,18)	95.52, < 0.001	7 (3,11)	97.54, < 0.001
Study quality	Good	4	15 (7,24)	97.75, < 0.001	10 (6,13)	91.12, < 0.001
	Very good	6	16 (12,20)	95.84, < 0.001	9 (5, 13)	97.83, < 0.001
Sample size	> 1000	4	16 (10,21)	97.61, < 0.001	8 (3,12)	98.44, < 0.001
	< 1000	6	16 (10,22)	96.46, < 0.001	11 (8,14)	88.89, < 0.001
Health sector transformation plan II	After	4	18 (15,22)	83.94, < 0.001	12 (10,13)	67.77, 0.03
	Before	6	14 (10,18)	96.41, < 0.001	8 (5,12)	97.07, < 0.01
COVID-19 era	Before	4	16 (9,22)	97.78, < 0.001	9 (3,15)	98.04,<0.001
	After	6	16 (12,20)	95.27, < 0.001	10 (7,12)	92.72, < 0.001
Adolescent age	10–19 year	2	11 (10,13)	93.3,<0.001	5 (1,9)	94.43, < 0.001
	10–24 year	8	17 (12,22)	97.5, < 0.001	11 (8,13)	93.3,<0.001
Publication year	< 5 years	6	16 (12,20)	95.27, < 0.001	10 (7,13)	92.72,<0.001
	> 5 years	4	16 (9,22)	97.78, < 0.01	9 (3,15)	98.04,<0.001
Overall		10	16 (12,19)	96.63, < 0.01	10 (6,13)	97.42, < 0.001

### Sensitivity analysis

**Fig. 4:**
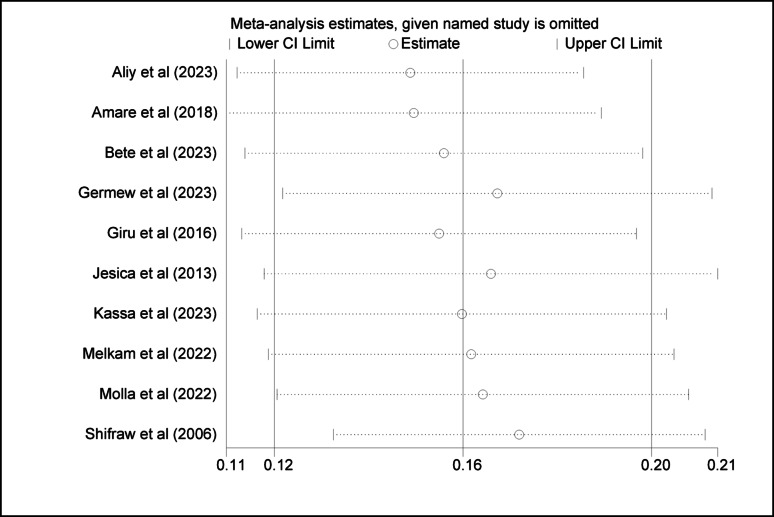
Sensitivity analysis of suicidal ideation among high school adolescents

**Fig. 5:**
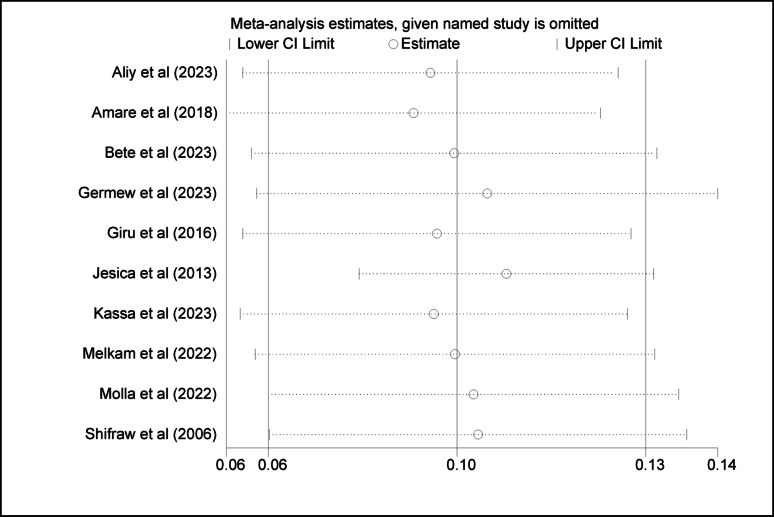
sensitivity analysis of suicide attempt among high school students

To assess the robustness of the magnitude of suicide attempts and ideation among adolescent results, we carried out sensitivity analysis by repeating and removing one study at a time and recalculating the summary of the effect size. The summary effect size remained constant, showing that our results were not determined by any single study (Figs. 4 and 5).

### Publication bias for suicidal ideation

**Fig. 6:**
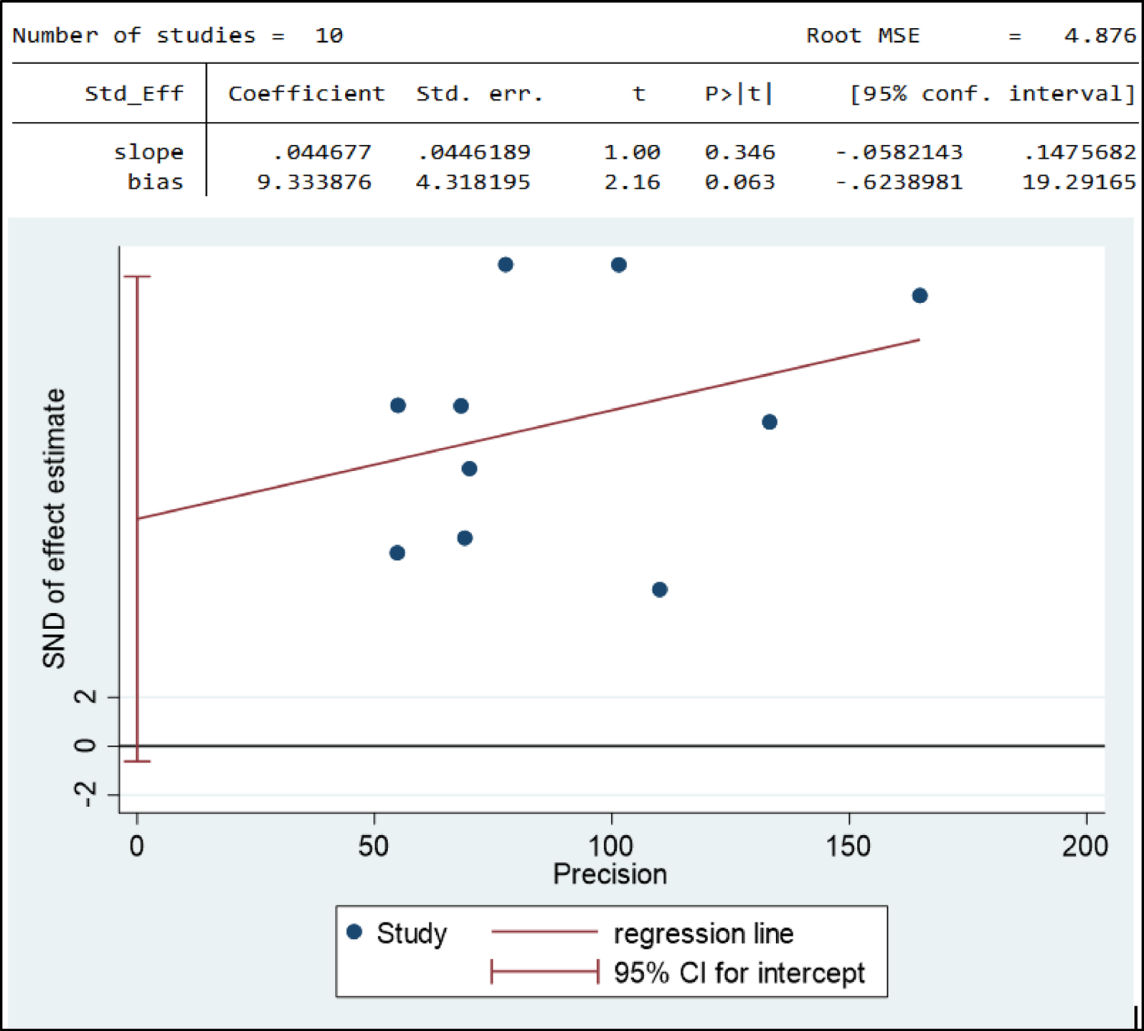
Funnel plot and egger test of publication bias for suicidal ideation

Funnel plot asymmetry was used to check the presence of publication bias (Fig. 12). The result of the funnel plot showed that there was a slight symmetrical distribution of articles. To confirm this asymmetry, we conducted an Egger test. The result of the Egger test showed that there was no statistically significant publication bias (p-value = 0.81) in estimating the prevalence of suicidal ideation among high school students in Ethiopia (Fig. 6).

### Publication bias for suicide attempt

**Fig. 7:**
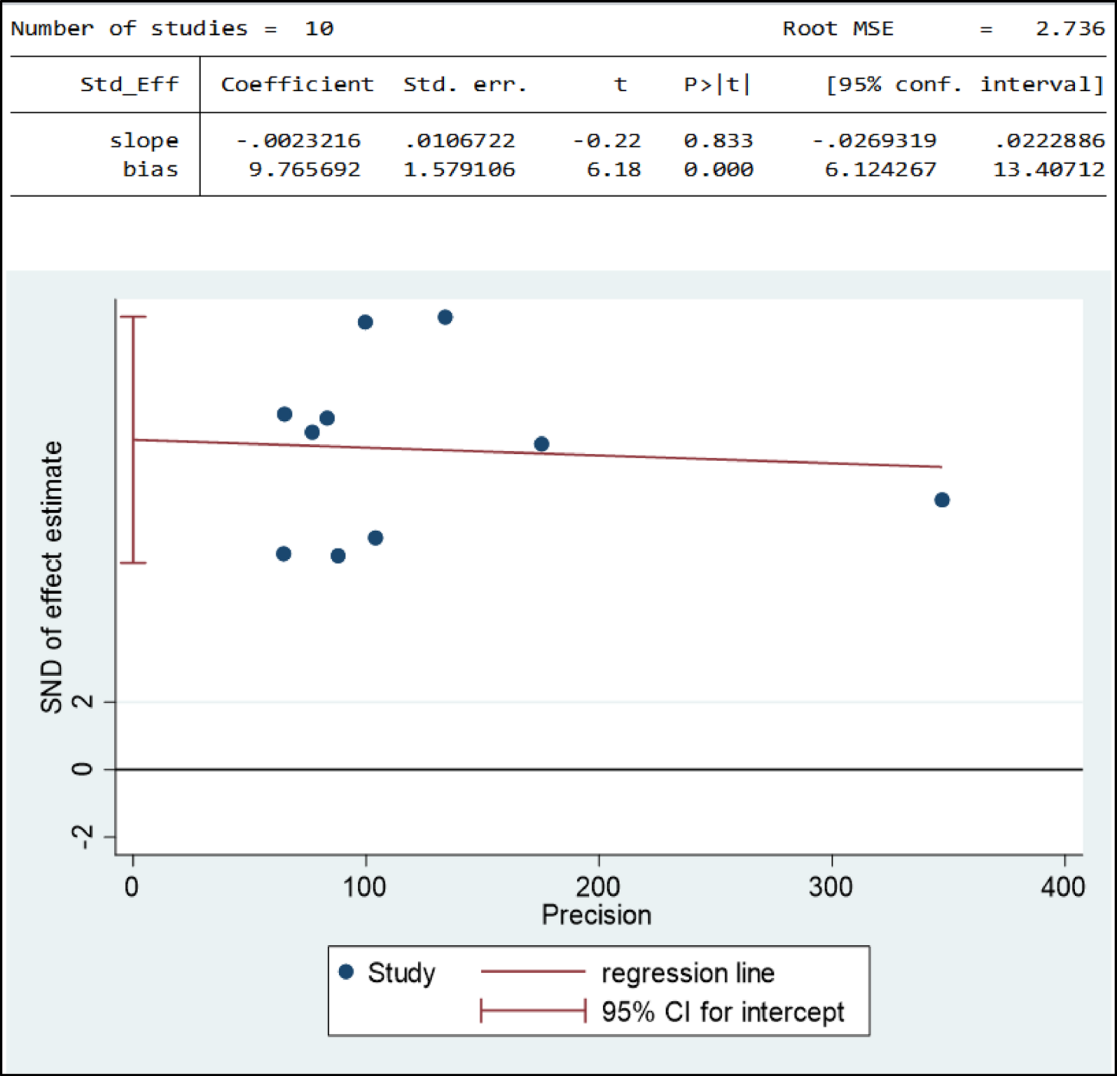
Funnel plot and egger test of publication bias for suicide attempts

The small-study effect refers to the tendency for estimates of intervention effects to be more beneficial in smaller studies. The estimated bias coefficient, or intercept, is 9.77, with a standard error of 1.579106. These standard errors reflect the uncertainty associated with our estimate of the bias coefficient. A larger standard error suggests greater uncertainty, while a smaller error indicates more precise estimates. The p-value with the bias coefficient is 0.000. A p-value less than 0.05 indicates that the bias coefficient is statistically significant. Based on test results, there is the presence of a small-study effect. Eventually, the selection biases introduced was fixed by doing trim and fill analysis. Accordingly, a non-parametric left funnel trim and fill analysis was done, and one imputed study was found. Finally, the study was excluded, and the final funnel plot is presented below (Fig. 7).

### Meta-trim and fill analysis for suicide attempt among high school adolescents

**Fig. 8:**
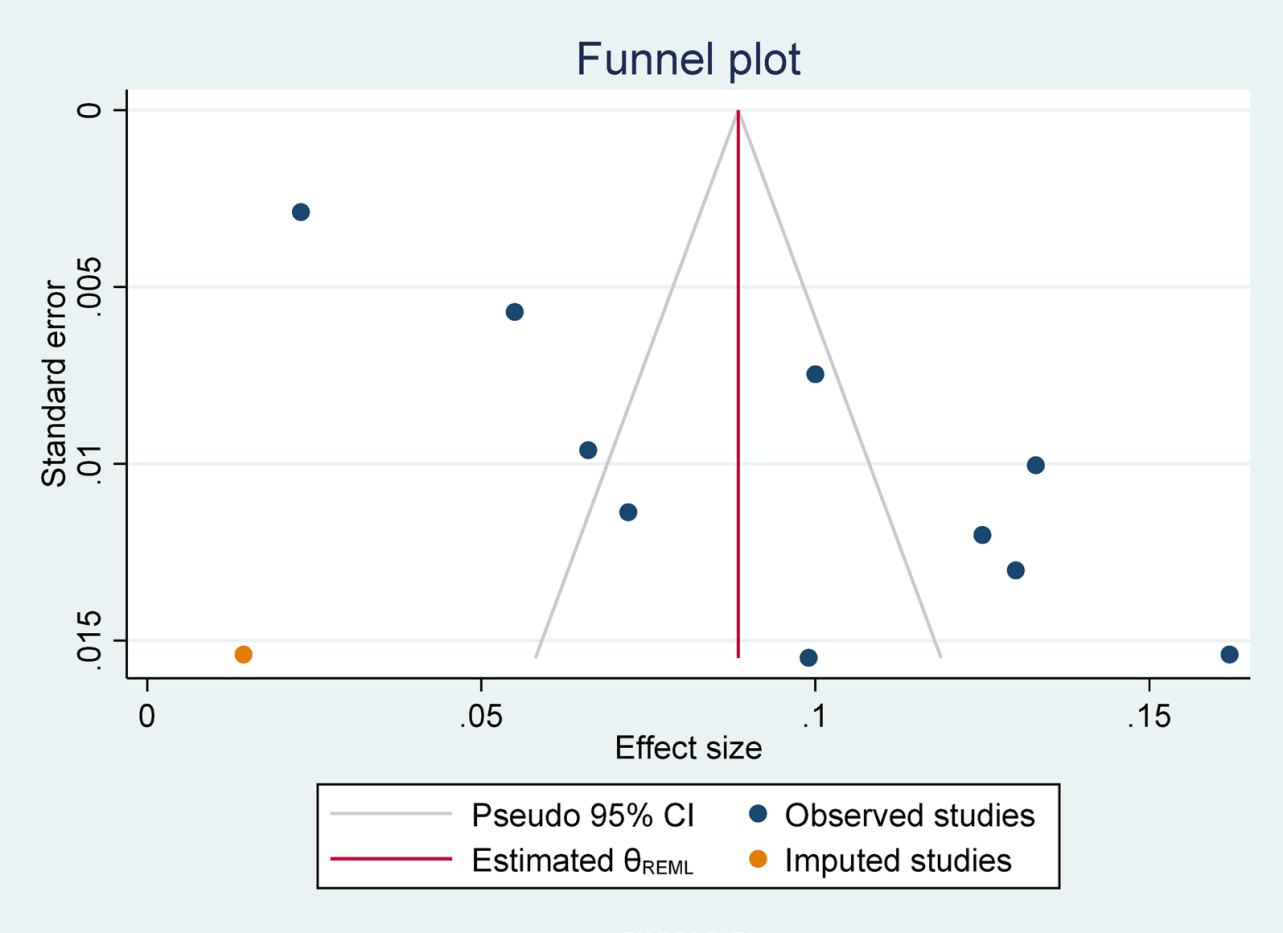
Meta-trim and analysis for suicide attempts among high school adolescents

To assess publication bias, a meta-trim and fill analysis was conducted. Both right and left trim-and-fill analyses were performed. The right trim-and-fill analysis did not identify any imputed studies, whereas the left trim-and-fill analysis revealed one imputed study, indicating potential bias as visualized in the accompanying figure (Fig. 8).

### Doi plot analysis for publication bias

**Fig. 9:**
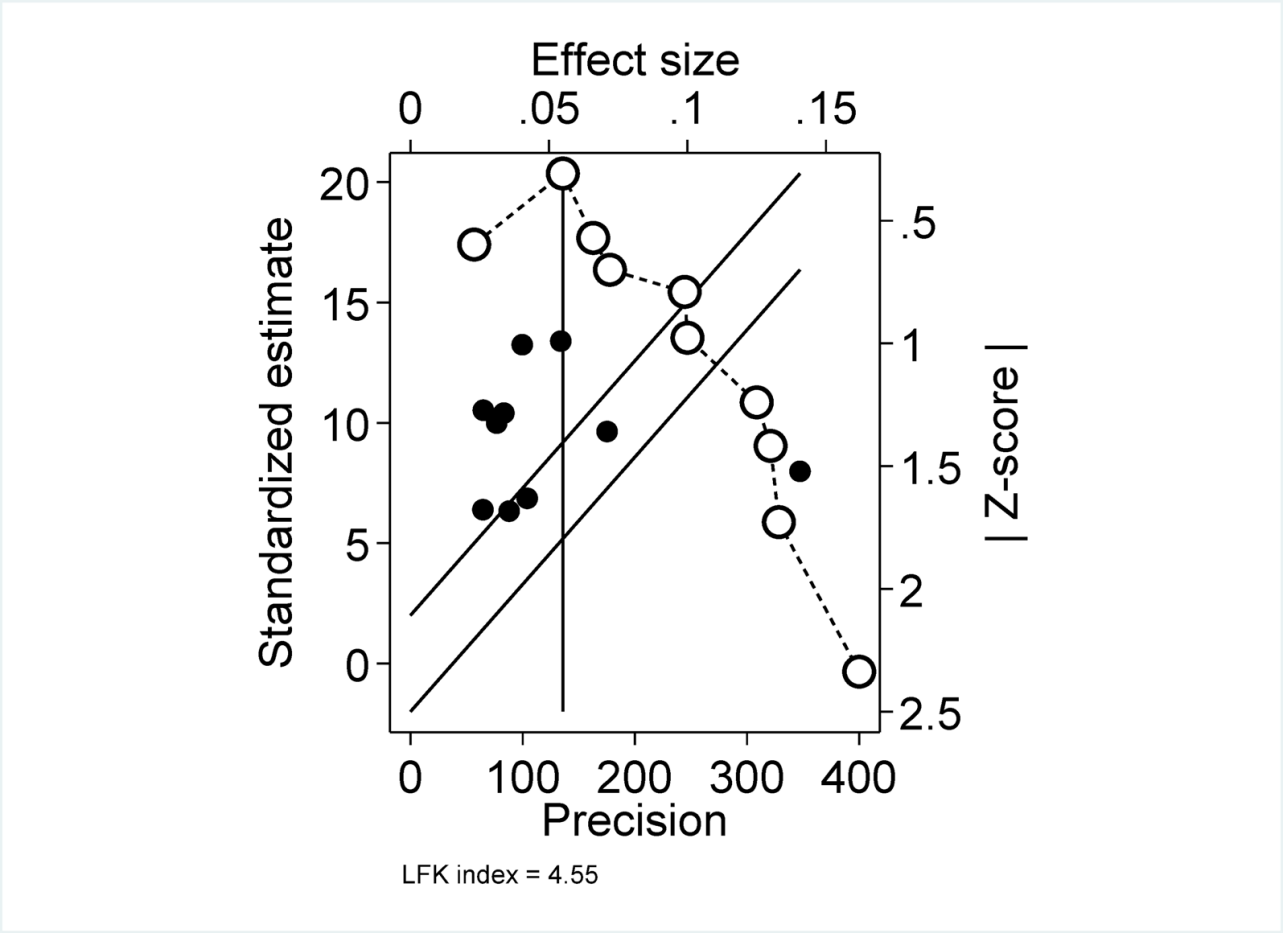
A Doi plot analysis for publication bias of studies reporting the prevalence of suicide attempt in Ethiopia

When a bias is present, visual asymmetry of a conventional funnel plot was usually used. However, concerns have been raised about both the visual appearance of funnel plots and the sensitivity of Egger’s regression to detect such asymmetry, particularly when the number of studies is small. As a result, we have used a Doi plot to better visualize asymmetry and also a new measure, the LFK index, to detect and quantify asymmetry of studies in Doi plots. The LFK index is a better discriminatory test between asymmetry between stimulated publication biases versus chance due to better area under the receiver operating characteristics curve and higher sensitivity over funnel plot. In our Doi plot analysis, asymmetrical distribution of studies suggests publication bias, and the LFK value of 4.8 lies outside − 1 and 1, indicating that there is asymmetry [47] (Fig. 9).

### Factors associated with suicidal ideation among high school adolescents

A vast array of variables was analyzed to see the association in order to identify the determinants of suicidal ideation among high school adolescents. Accordingly, gender, disappointment with school results, alcohol use, family history of suicide attempt, presence or absence of social support, history of abuse, living arrangement, anxiety, and depression were included to see the association with suicidal ideation.

#### The association between female gender and suicidal ideation among high school adolescents

**Fig. 10:**
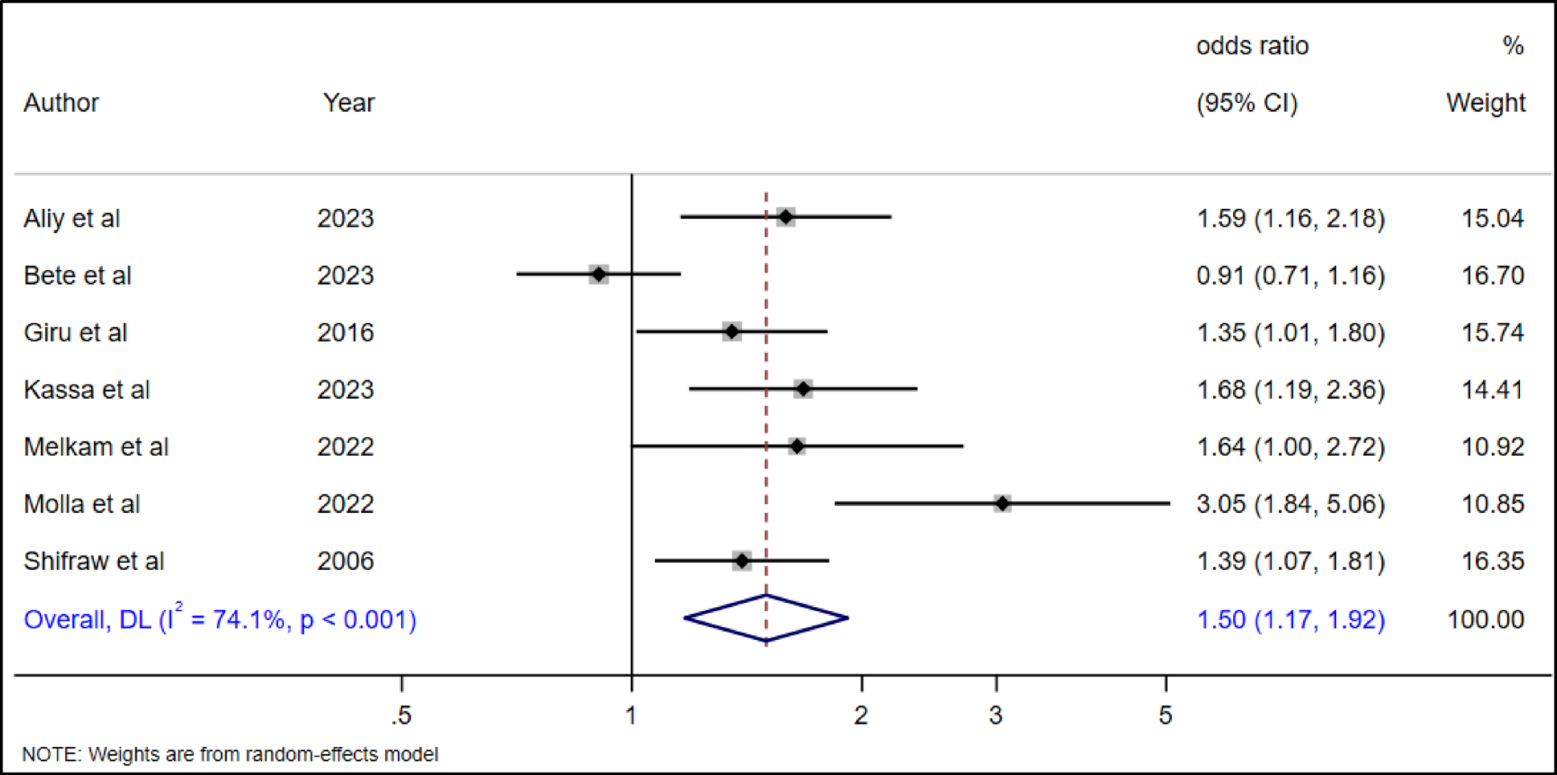
The association between female gender and suicidal ideation among adolescents

To examine the association between gender and suicidal ideation, seven (*n* = 7) studies were included in the analysis [19, 28, 29, 35, 36, 38, 39]. A random effect model was used to estimate the pooled association between gender and suicidal ideation among high school adolescents (I²=74.71, p-value < 0.001). Our pooled analysis revealed that female adolescents had 50% higher chances of suicidal ideation (AOR = 1.5, 95% CI 1.17–1.92) as compared to male adolescents (Fig. 10).

#### The association between school result and suicidal ideation among high school adolescent

**Fig. 11:**
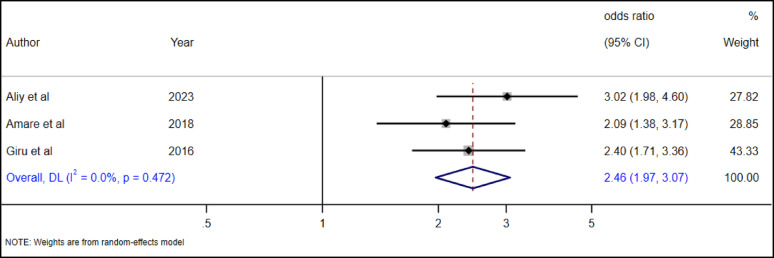
The association between disapponting school result and suicidal ideation among adolescents

Three studies [28, 34, 36] were included to examine the association between whether disappointing school results were associated with suicidal ideation or not. Our pooled analysis revealed that female adolescents had 2.46 times higher odds of suicidal ideation (AOR = 2.46, 95% CI 1.97–3.07) as compared to male adolescents (Fig. 11).

#### The association between family history of suicide attempts and suicidal ideation among high school adolescents

**Fig. 12:**
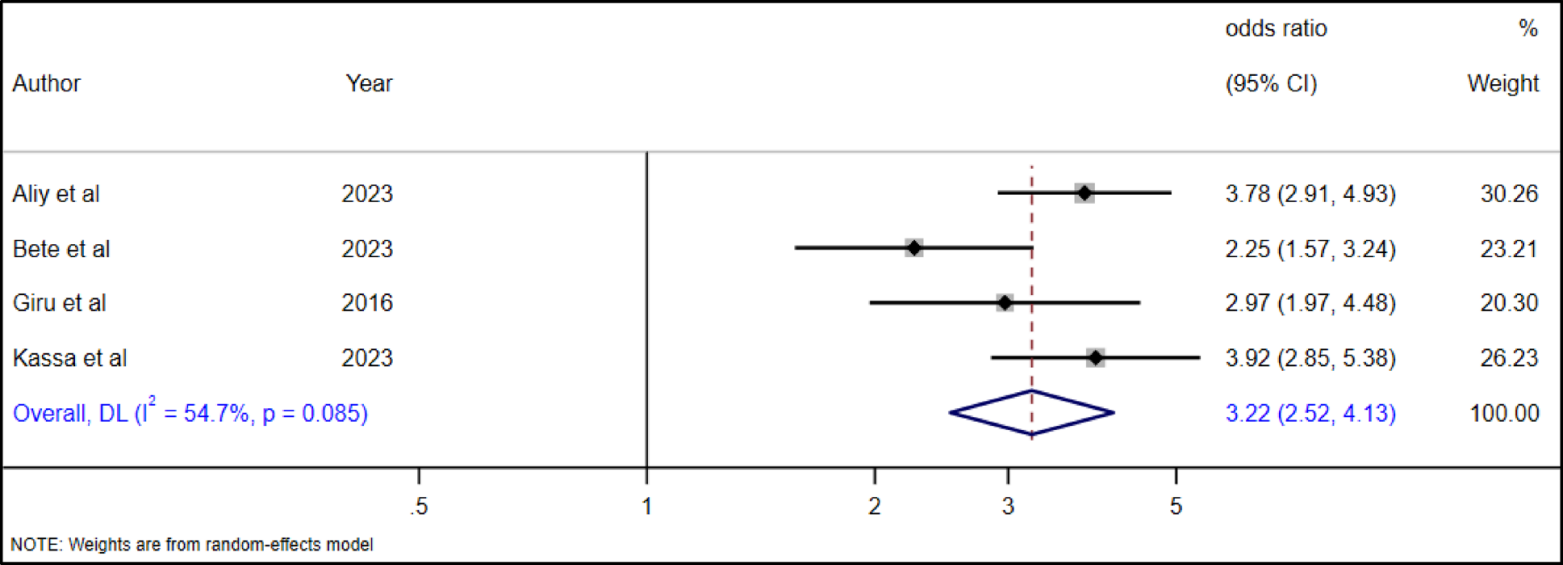
The association between family history of suicide attempts and suicidal ideation among adolescents

The association between suicidal ideation and family history of suicide attempts was investigated with four studies [19, 28, 35, 36]. The pooled random effect estimate showed that adolescents with a family history of suicidal tendency had 3.22 times higher likelihood of developing suicidal ideation than adolescents from parents without suicidal history (Fig. 12).

#### The association between alcohol use and suicidal ideation among high school adolescents

**Fig. 13:**
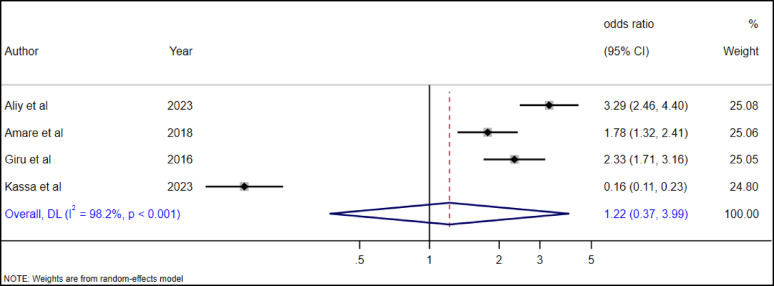
The association between alcohol use and suicidal ideation among high school adolescents

Alcohol use is a common practice among high school adolescents in Ethiopia. For instance, a study done in four major regions of Ethiopia and Addis Ababa revealed that 41.8% high school adolescents consumed alcohol in their lifetime [48]. Alcohol use among adolescents and suicidal ideation were assessed by four studies [19, 28, 34, 36]. Nevertheless, no single study or pooled estimate showed a significant association between alcohol use and suicidal ideation among high school adolescents in Ethiopia (AOR = 1.22, 0.37–3.99) (Fig. 13).

#### The association between social support and suicidal ideation among high school adolescents

**Fig. 14:**
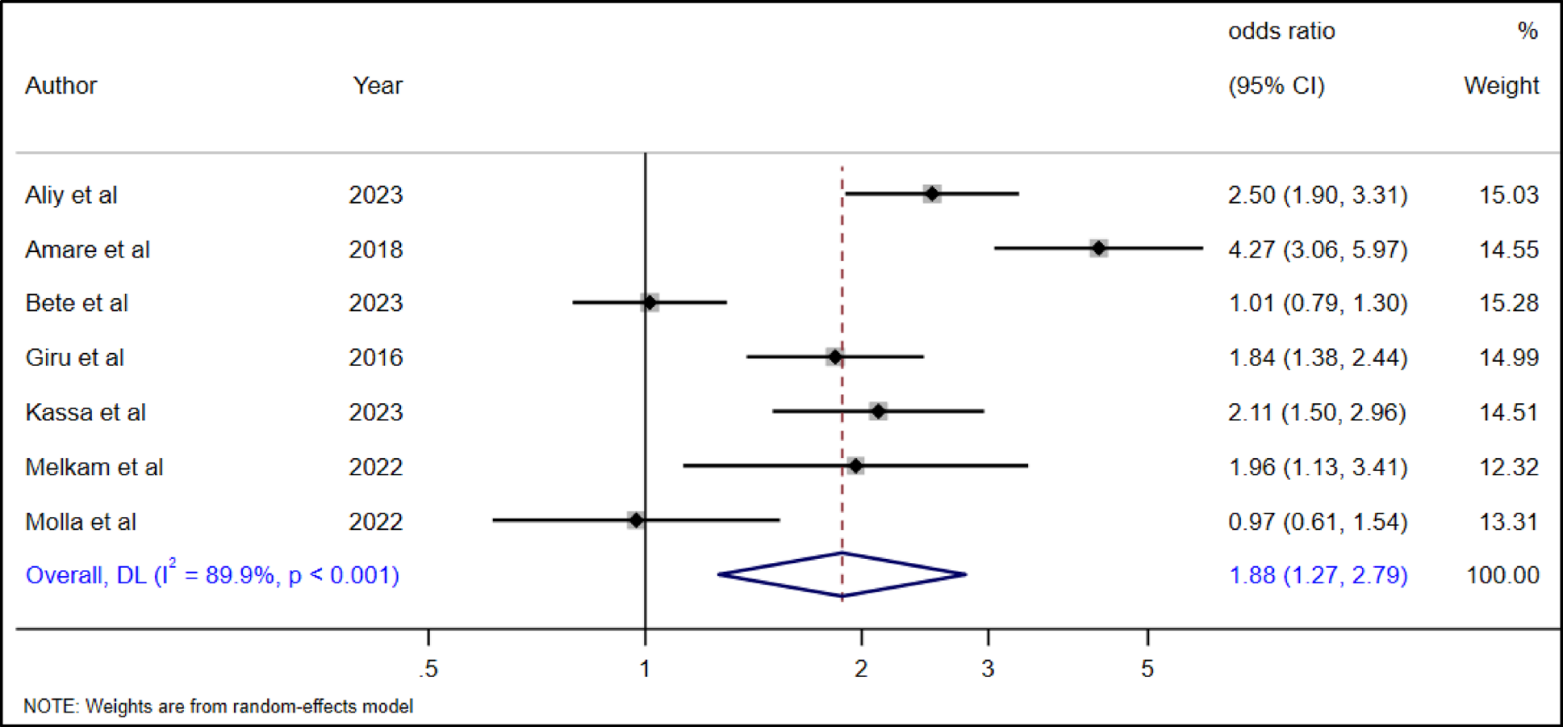
The association between social support and suicidal ideation among high school adolescents

The association between social support or family support and suicidal ideation was investigated in seven studies [19, 28, 29, 34–36, 39]. The pooled estimate showed that adolescents without family or social support had an 88% more likely (AOR = 1.88, 95% CI: 1.27–2.79) chance of suicidal ideation as compared to adolescents with family or social support (Fig. 14).

#### The association between anxiety and suicidal ideation among high school adolescents

**Fig. 15:**
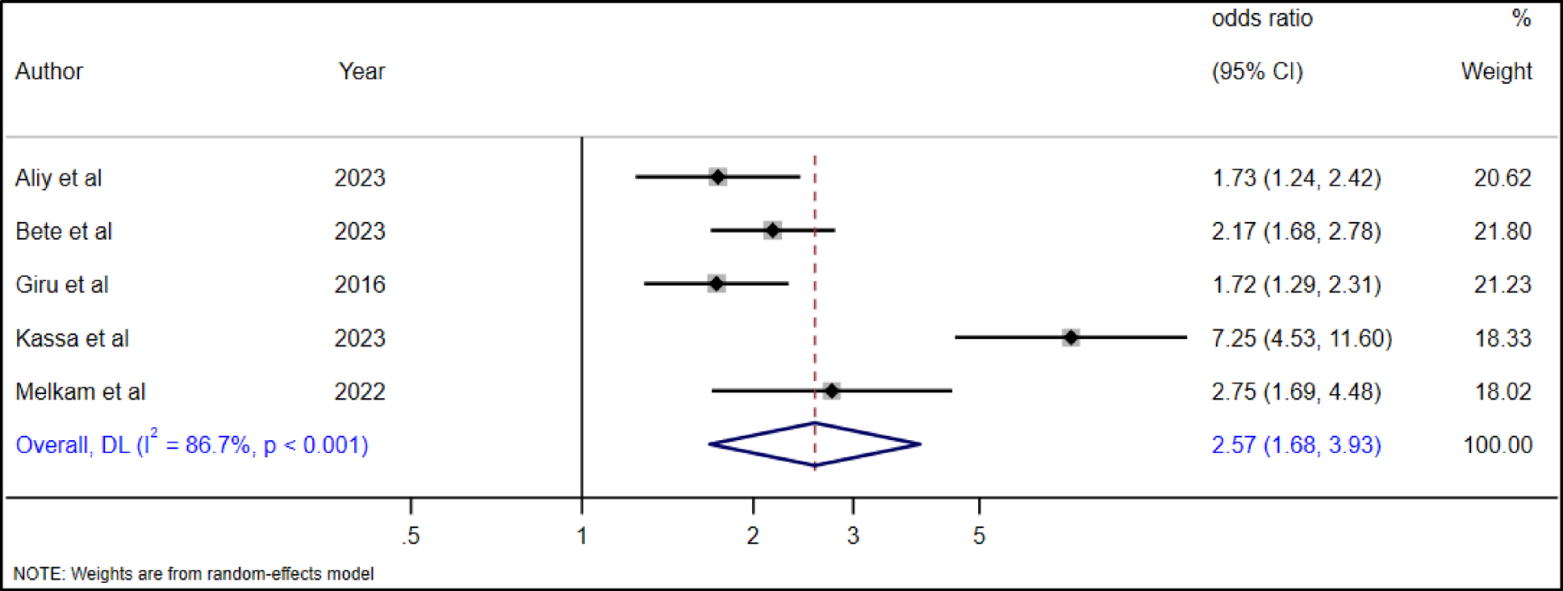
The association betweeen anxiety and suicidal ideation among highschool adolsecents

To examine the association between anxiety and suicidal ideation among high school adolescents, five studies were included [19, 28, 35, 36, 38]. The overall pooled estimate revealed that adolescents with anxiety had 2.57 (AOR = 2.57, 95% CI: 1.68–3.93) higher odds of suicidal ideation as compared to adolescents without anxiety (Fig. 15).

#### The association between depression and suicidal ideation among high school adolescents

**Fig. 16:**
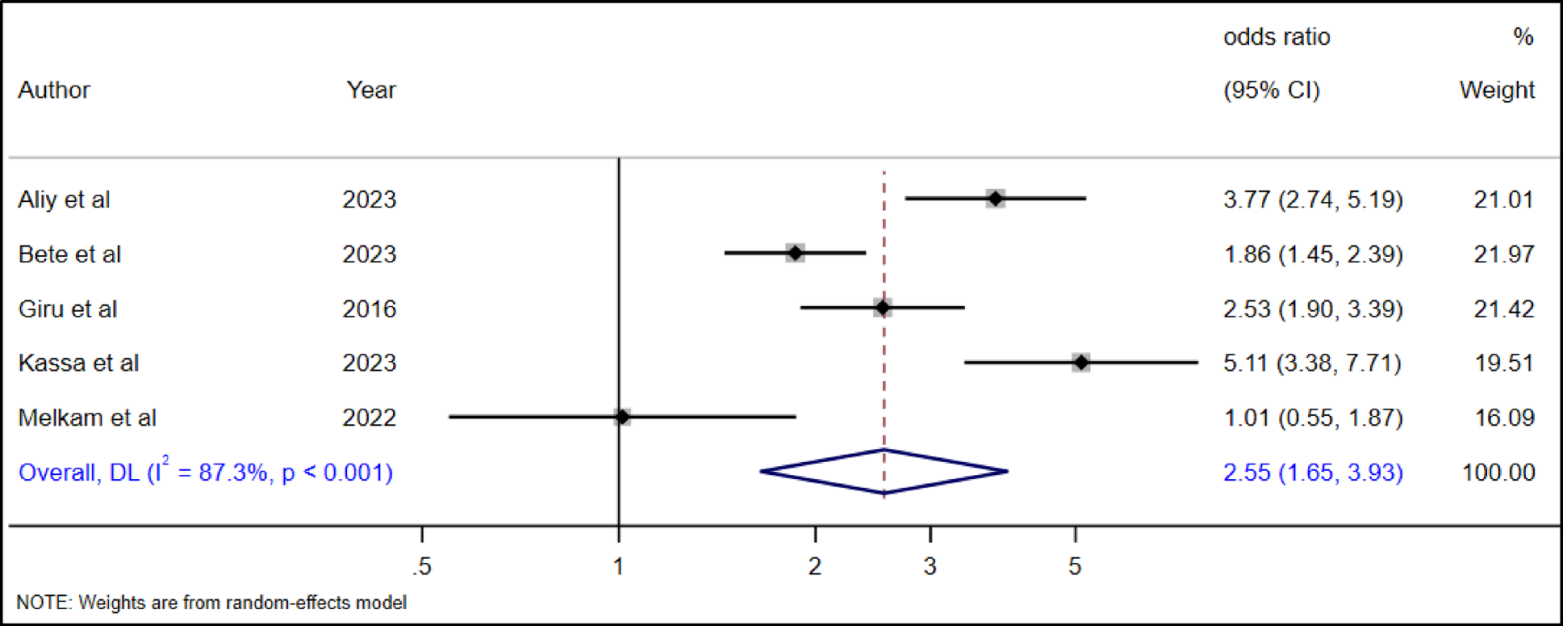
The association between depression and suicidal ideation among high school adolescents

The association between depression and suicidal ideation was examined in five studies [19, 28, 35, 36, 38]. The overall random effect pooled estimate revealed that adolescents with depression had 2.55 (AOR = 2.55, 95% CI, 1.65–3.93) times higher odds of suicidal ideation than adolescents without depression (Fig. 16).

#### The association between living arrangement of adolescent and suicidal ideation

**Fig. 17:**
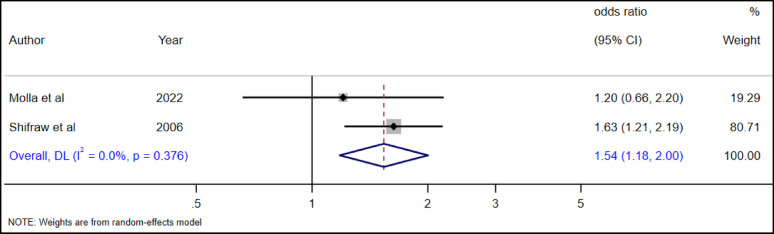
The association between living arrangement and suicidal ideation among adolescents

Two primary studies [29, 39] that examined the association between suicidal ideation and living arrangements among adolescents were included in our meta-analysis. In our review we found a significant association between living arrangement and suicidal ideation among adolescents. Accordingly, adolescents living without their parents had a 54% (AOR = 1.54, 95% CI: 1.18-2.00) more likely chance of suicidal ideation as compared to adolescents living with their parents (Figure 17).

#### The association between adolescent abuse & suicidal ideation

**Fig. 18:**
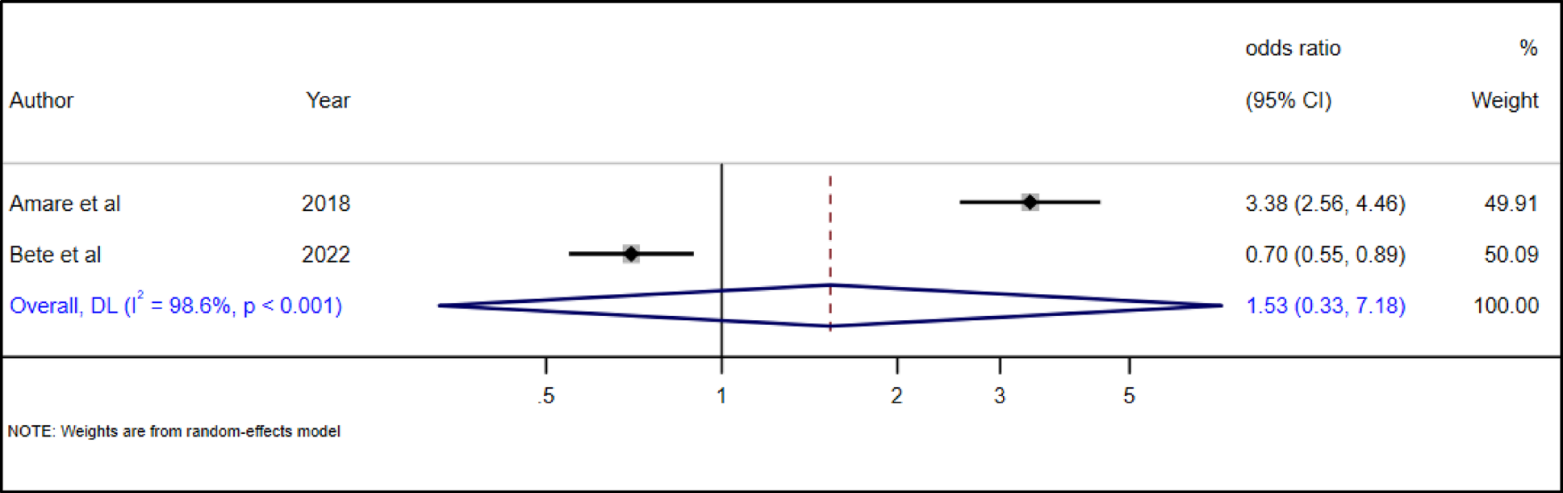
The association between adolescent abuse & suicidal ideation among adolescents

The association between adolescent history of abuse and suicidal ideation was examined in two primary studies [34, 35]. In our review we found that there is no statistically significant association (AOR = 1.53, 95% CI: 0.33–7.18) between any kind of adolescent abuse history and suicidal ideation (Fig. 18).

### Factors associated with suicide attempts among high school adolescents in Ethiopia

To see the association between suicide attempts and different factors, including gender, disappointment with school results, alcohol use, family history of suicidal attempts, presence or absence of social support, anxiety, and depression among adolescents, were included in this review.

#### The association between female sex and suicide attempts among high school adolescents

**Fig. 19:**
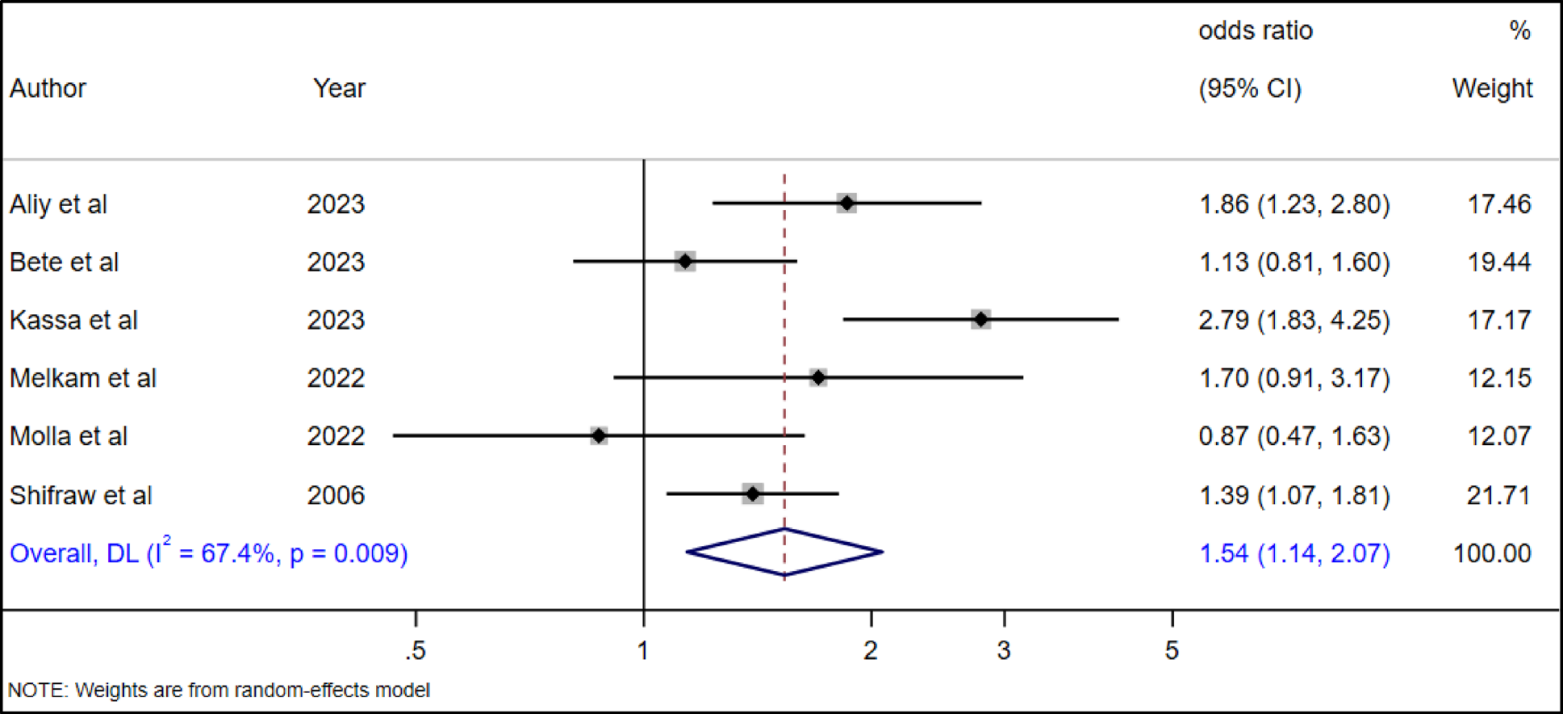
The association between female gender and suicide attempts

The association between female gender and suicide attempts was examined in six studies [19, 28, 29, 35, 38, 39] in our meta-analysis. Our pooled random effect estimate revealed female adolescents had a 54% more likely (AOR = 1.54, 95% CI: 1.14–2.07) chance of dying by suicide as compared to male adolescents (Fig.19).

#### The association between school result and suicide attempts among high school adolescents

**Fig. 20:**
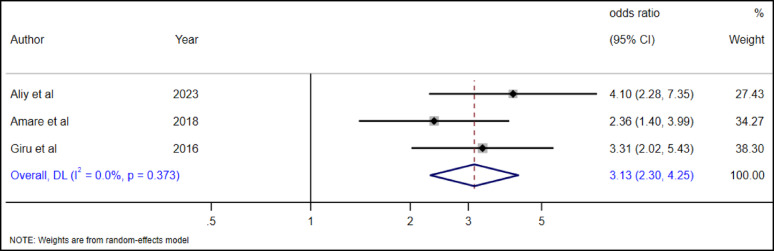
The association between school result and suicide attempt among high school adolescents

To examine the association between disappointment with school results and suicide attempts, three studies were included [28, 34, 36]. Our overall pooled estimate revealed there is no significant association (AOR = 2.53, 95% CI: 0.63–10.08) between disappointment with school results and suicidal attempts (Fig. 20).

#### The association between family history of suicide attempts and high school adolescent suicide attempt

**Fig. 21:**
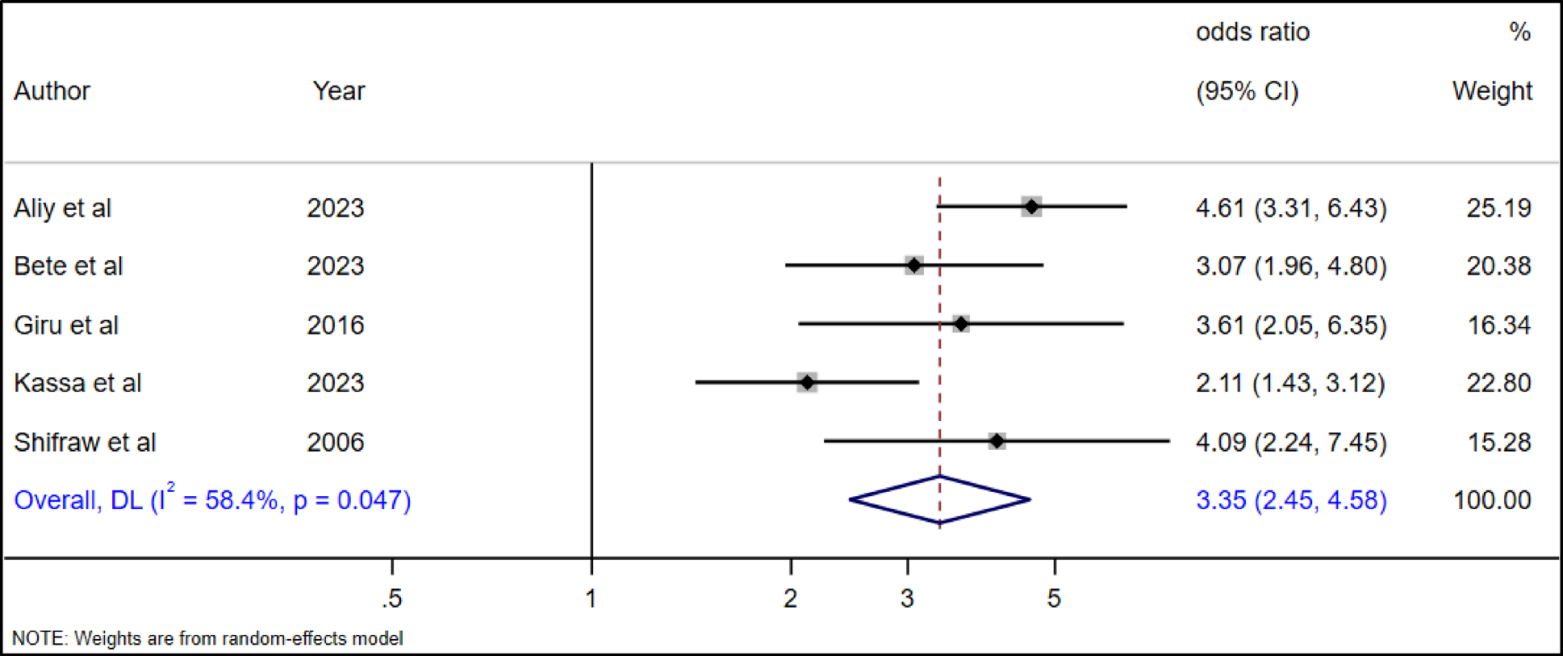
The association between family history of suicide attempt and suicide attempts among high school adolescents

The association between family history of suicide attempts and the adolescent’s own attempts was examined in five studies [19, 28, 29, 35, 36]. The overall pooled estimate revealed a significant association between family history of suicide attempt and adolescent suicide attempt (AOR = 3.35, 95% CI, 2.45–4.58). Accordingly, adolescents with a familial suicide attempt were 3.35 times more likely to have had a suicide attempt as compared to adolescents without a familial history of suicide attempts (Fig. 21).

#### The association between alcohol use and suicide attempts among high school adolescents

**Fig. 22:**
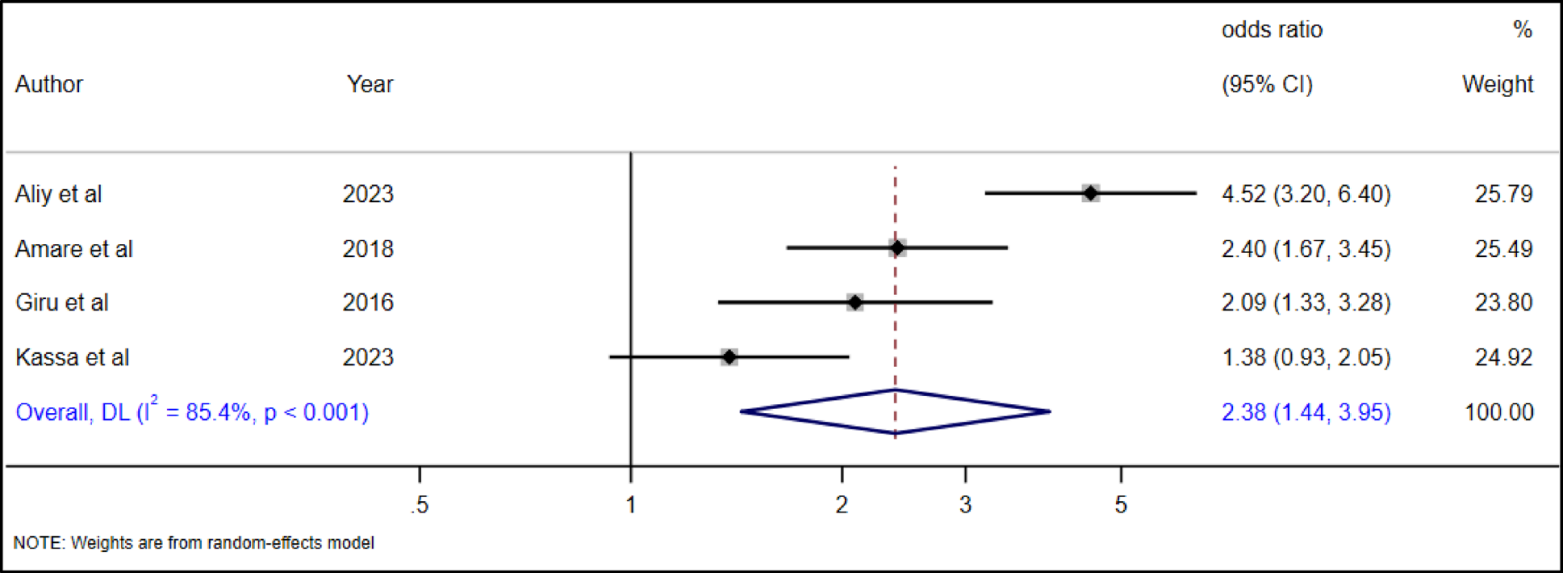
The association between alcohol use and suicide attempt among high school adolescents

Alcohol and other substance use are commonly practiced in Ethiopia. A systematic review and meta-analysis report indicated that 27.67% of adolescents used alcohol in Ethiopia [49]. As a result of seeing the association with suicide attempts, we included four primary studies reporting the association between alcohol use and suicide attempts [19, 28, 34, 36]. According to the random effect pooled analysis, adolescents who have consumed alcohol had 2.38 times (AOR = 2.38, 95% CI: 1.44–3.95) higher odds of suicide attempts than adolescents without alcohol use (Fig. 22).

#### The association between family/social support and suicide attempt among high school adolescents

**Fig. 23:**
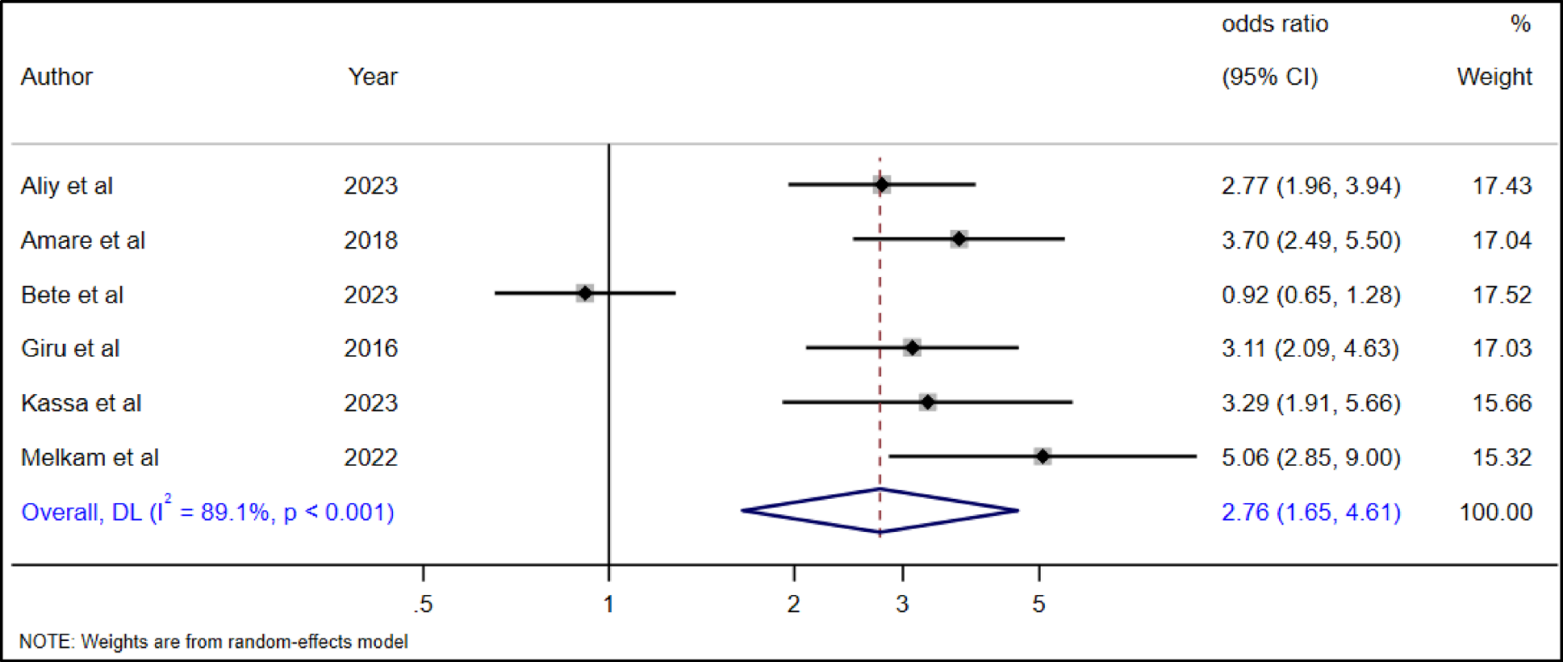
The association between absence of social support and suicide attempt among high school adolescents

Six studies [19, 28, 34–36, 38, 39] were included to see the association between no social support and suicide attempts among adolescents in Ethiopia. The overall pooled random effect estimate showed that adolescents without family/social support had 2.76 times higher odds of suicide attempts (AOR = 1.90, 95% CI 1.65–4.61) as compared to adolescents with family or social support (Fig. 23).

#### The association between anxiety and suicide attempt among high school adolescents

**Fig. 24:**
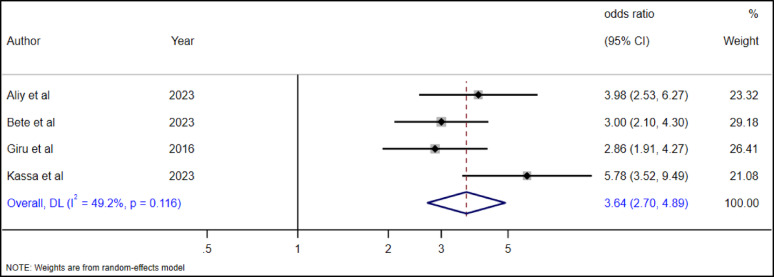
The association between anxiety and suicide attempt among high school adolescents

The association between anxiety and suicide attempts among high school adolescents was examined with four studies [19, 28, 35, 36]. According to the overall pooled random effect estimate, adolescents with anxiety had 3.64 times higher (AOR = 3.64, 95% CI: 2.7–4.89) odds of suicide attempts as compared to adolescents without anxiety (Fig. 24).

#### The association between depression and suicidal attempt among high school adolescents

**Fig. 25:**
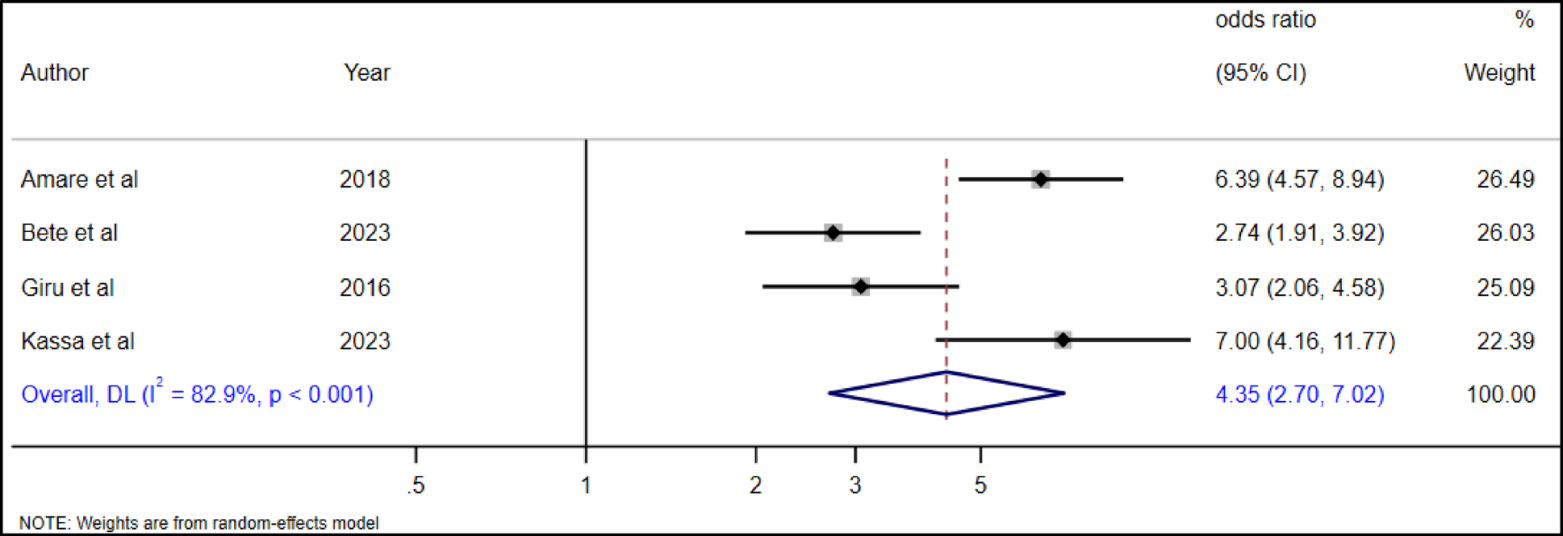
The association between depression and suicide attempt among high school adolescents

The association between depression among high school adolescents and suicide attempts was examined by six studies [19, 28, 34–36]. The overall pooled estimate of our analysis also revealed a significant association between depression and suicide attempts. Accordingly, adolescents with depression have 4.35 times higher odds (AOR = 4.35, 95% CI: 2.7–7.02) of attempting suicide as compared to adolescents without depression (Fig. 25).

#### The association between living arrangement and suicide attempt

**Fig. 26:**
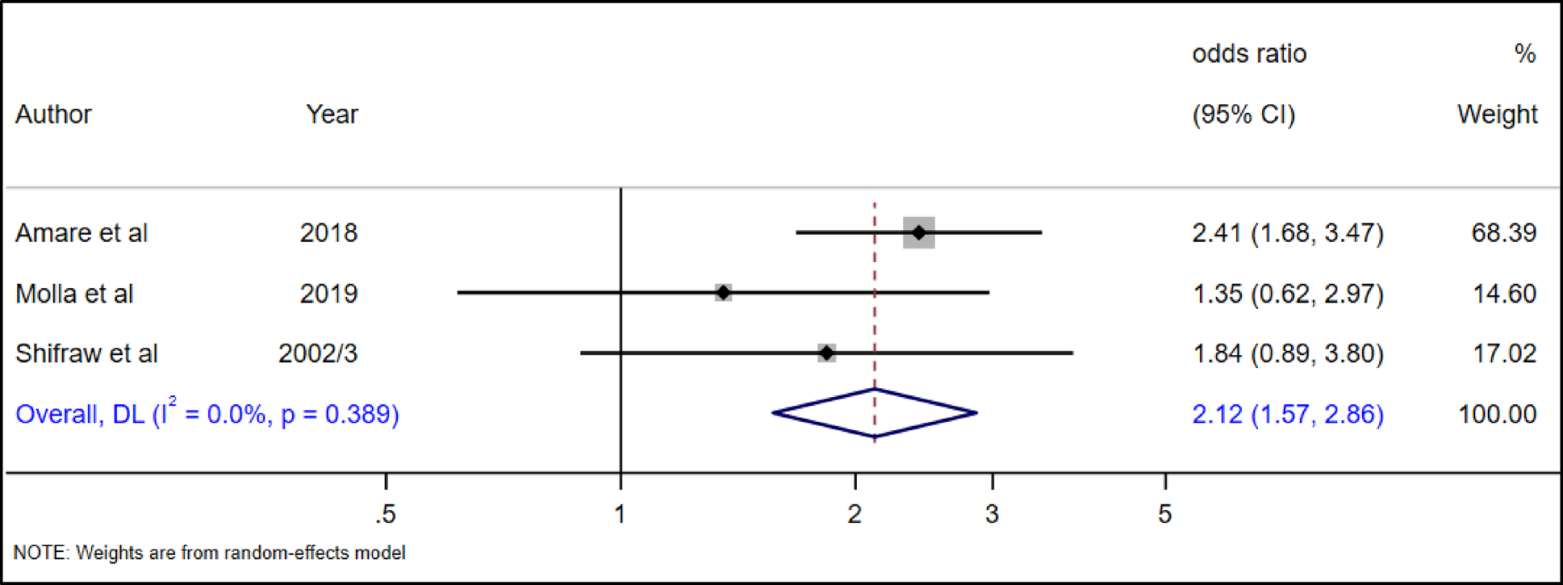
The association between living arrangement of adolescent and suicide attempt

The association between the living arrangement of adolescents and suicide attempts was addressed in three primary studies [29, 34, 39]. Our meta-analysis found that adolescents living without their parents had 2 times (AOR = 2.12, 95% CI: 1.57–2.86) higher odds of suicide attempts as compared to adolescents living with their parents (Fig. 26).

#### The association between adolescent abuse history and suicide attempt

**Fig. 27:**
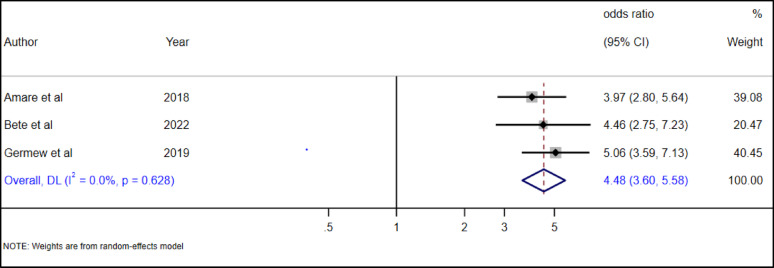
The association between adolescent history of abuse and suicide attempt

Three studies [30, 34, 35] examined the association between adolescent history of any abuse and suicide attempts. Our pooled meta-analysis estimate revealed that adolescent with abuse history had 4.48 times (AOR = 4.48, 95% CI: 3.6–5.58) higher odds of suicide attempt as compared to adolescent without any abuse history (Fig. 27).

## Discussion

The findings of this study reveal a significant prevalence of suicidal ideation and suicide attempts among high school adolescents in Ethiopia, underscoring the need to address mental health challenges within this vulnerable population. Despite the Ethiopian government’s commitment to mental health care through the Health Sector Transformation Plan II and the national mental health strategy, our results indicate gaps in understanding the scope and determinants of these issues. Accordingly, this systematic review found the pooled prevalence of suicidal ideation among high school adolescents was 16% (95% CI, 12-19%). This highlights the need for more targeted interventions and robust policies to reduce suicidal intention in adolescents.

This study finding is consistent with the study done in 59 low- and middle-income countries (16.9%) and the study done on 82 countries [50, 51]. The possible explanation for consistency with findings in low- and middle-income countries may lie in shared socio-economic and cultural challenges, such as poverty, limited access to mental health services, stigma surrounding mental health, and social pressures that contribute to suicidal ideation. Additionally, the growing impact of globalization and cyberbullying could further explain the observed alignment across these regions [52, 53].

However, this study finding is lower than the studies done in Ghana (18.2%) [54] and Iran (21%) [55]. The difference might be due to methodological differences like sample size, cultural and social dynamics, government intervention, and priorities made on adolescent mental health.

This review found the pooled prevalence of suicide attempts among high school adolescents was 10% (95% CI, 6-13%). This review finding was comparable with the study done in Canada (10.9%) [56]. This review finding is higher than the study done in China (2.94%) [57] and Vietnam (3.8%) [58]. This shows that suicidal behavior among high school students may be a global issue that crosses geographical and cultural boundaries. The disparity may be due to differences in cultural views, mental health care availability, socioeconomic situations, and mental health reports. However, this review finding is lower than the study finding from Iran (18%) and low-and middle-income countries (17.0%) [50, 55]. The discrepancy in findings might result from variations in economic, social, and cultural factors. Furthermore, the credibility, availability, or lack of reliable data and cultural nuances may result in different findings.

According to the review sub-group analysis, studies done after the mental health strategic plan had a higher pooled prevalence of suicidal ideation and suicide attempts than studies done before this strategic plan. The possible explanation for this highest finding might be increased awareness and better reporting since the National Mental Health Strategic Action Plan (NMHSAP) focuses on emphasis creation, awareness, community mobilization, and advocacy for mental health. In addition, NMHSAP focuses on expanding mental health services to improve access so that more individuals are seeking help and being diagnosed, thus increasing the prevalence rate [59]. Therefore, the government should strengthen these initiatives to address this emerging mental health problem. In addition, the government should make all efforts to successfully complete and implement the suicide prevention implementation research project in Ethiopia [21].

According to the subgroup analysis, the lowest pooled prevalence of suicidal ideation and suicide attempt was observed in early adolescents (10–15) compared to late adolescents (15–24). This might be due to early adolescents’ protective factors like strong parental supervision and lesser exposure to stressors as compared to older adolescents and youth, whereas older adolescents and young adults often face increased pressure related to unemployment, relationship breakup, identity struggle, financial independence challenges, and social isolation that heighten suicidal ideation and suicide attempts [60, 61].

Subgroup analyses were conducted based on study subjects with known risk factors for suicide, including studies done in female adolescents, studies done during and on the area (Amhara region) where the Ethiopian Tigray war took place, studies done in substance-using adolescents, and studies done in the study subjects without the above risk factors. Surprisingly, a lower rate of suicidal ideation and suicide attempt was observed in studies done with study subjects with risk factors. Accordingly, adolescents’ exposure to risk factors for suicide had a lower prevalence of suicidal ideation and suicide attempts (14%, at insignificant I² and p-value) and (7%, at insignificant I² and p-value), respectively. While the subgroup analysis made without any risk factor for suicide had a higher prevalence of suicidal ideation (17%, I² = 97.57, *p* < 0.001). One explanation would be because study subjects in war-affected areas frequently build resilience or coping mechanisms through cooperation and adaptability in dire circumstances [62]. Substance-using adolescents may also use drugs to temporarily numb their mental pain, postponing outward manifestations of suicidal thoughts and suicide attempts [63]. Additionally, female teenagers might take psychological therapy due to their stronger social networks and superior emotional expression.

Subgroup analysis was done by study setting, by which the highest prevalence of suicidal ideation and suicide attempts was observed in pure urban areas and the lowest prevalence of suicidal ideation and suicide attempts was observed in semi-urban areas. This discrepancy may be explained by the fact that urban adolescents face academic competition because of the high availability of educational opportunities and social expectations for success; higher living expenses and financial strain in urban areas; social division and lack of community bond; increased accessibility to substances and drugs; increased exposure to violence and crime; economic disparity; and peer pressure, all of which increase the likelihood of suicidal ideation and suicide attempts among urban adolescents [64, 65].

This meta-analysis was conducted using at least two articles that reported risk factors. These studies were analyzed through a random effect model. Accordingly, the risk factors analyzed were gender, disappointment with school results, family history, alcohol use, the presence or absence of family or social support, anxiety, and depression.

According to this meta-analysis, as compared to males, females were more likely to have suicidal ideation and suicide attempts. This is in line with another review [66, 67]. This might be due to biological, psychological, and social factors. Hormonal changes throughout puberty can influence mood and emotional regulation, making female adolescents more susceptible to anxiety and sadness, both of which are substantial risk factors for suicide [68]. Women also internalize emotion more than men. Women often engage in rumination (dwelling in emotion) more than men, while men may be more likely to use distraction or problem-solving strategies. Adolescent girls are frequently subjected to social expectations about relationships, beauty, and academic achievement; as a result of all the above reasons, they might expose themselves to self-harm and other unhealthy coping strategies [69–71].

In this review, adolescents without family or social support had a higher chance of having suicidal ideation and suicide attempts as compared to adolescents with supporters. This is supported by other studies [72, 73]. Adolescents without family or social support are more likely to feel isolated and overwhelmed, lack resources for mental health, lack emotional support during stress and anxiety, feel disconnected, and end up with maladaptive coping mechanisms like self-harm and suicide.

Adolescents with disappointing school results are significantly associated with suicidal ideation and suicide attempts. Accordingly, adolescents with disappointing school results had higher odds of suicidal ideation and suicide attempts [74]. This is due to the fact that academic underachievement has a significant impact on self-esteem and future prospects, and bad results can lead to emotions of inadequacy, embarrassment, and fear of judgment. In societies that place a strong emphasis on academic success, family expectations create additional stress, and without proper mental health support, individuals may become more susceptible to extreme reactions, including suicidal ideation and suicide attempts.

In the pooled meta-analysis, family history of suicidal ideation and suicide attempts was positively associated with suicidal ideation and suicide attempts among adolescents. This is in line with other study’s [75–77]. This might be due to an adolescent who witnessed their parent using a suicidal thought and trial as a coping mechanism for distress may have come to perceive it as normal. In addition, parental impulsive aggression predisposes individuals to family instability and abuse, which increases the risk of suicidal behavior in children [78]. Furthermore, suicidal behavior aggregate in families, and both genetic and non-genetic factors responsible for familial transmission [79].

Alcohol use by adolescents is significantly associated with suicide attempts. This is supported by other studies [80, 81]. This is due to the fact that alcohol intoxication can increase maladaptive cognitive behavior and hinder self-regulation, thereby increasing the risk of suicide. In addition, alcohol use can reduce a person’s awareness of their negative emotions and impair their ability to use healthy coping skills to manage stressful situations [80].

Adolescents who had anxiety had higher odds of suicidal ideation and suicide attempts as compared to adolescents without anxiety. This is supported by other studies [82–86]. This is due to the fact that anxiety often causes persistent worry, fear, and emotional turmoil; over time these feelings lead to despair and hopelessness. Anxiety promotes rumination, repetitive negative thinking that escalates into thoughts of self-harm and suicide, and a high level of anxiety can impair a child’s ability to cope with everyday stressors, creating a sense of inadequacy and failure [87]. Anxious adolescents often avoid social interaction due to fear of judgment or rejection. This isolation can lead to loneliness, a known risk factor for suicidal ideation and suicide attempts. Children with anxiety may misinterpret or undervalue the support they receive, believing they are a burden to others, which heightens feelings of worthlessness. Anxiety often manifests as physical symptoms, e.g., headaches, which can further distress adolescents, making them feel overwhelmed or helpless [88, 89].

Adolescents who had depression were more likely to have suicidal ideation and suicide attempts than adolescents without depression. This is supported by the other studies [74, 75, 90]. This is due to the fact that depressed adolescents had emotional instability, cognitive distortion, altered thinking, and diminished ability to cope with stress [91, 92]. It is also indicated that decreased levels of the serotonin neurotransmitter in the brains of depressed individuals were found to be associated with suicidal attempts [93]. In addition, adolescents may experience symptoms like hopelessness, helplessness, giving up, and feelings of isolation and despair, which were the core features of demoralization, which in turn leads to suicidal attempts [94]. Therefore, early identification and treatment through mental health screening, strengthening the support network, teaching coping skills, and increasing mental health education are key interventions to halt the problem.

Adolescents living without their biological parents had higher odds of suicidal ideation and suicide attempts as compared to adolescents living with their biological parents. This is supported by other studies [95–97]. This is due to the fact that adolescents living without biological parents always face lower self-confidence, heightened anxiety and loneliness, more depressed moods, and a lack of emotional support and guidance [96, 98]. To address the challenges faced by these adolescents, it is crucial to establish community-based programs that offer consistent emotional and social support. Additionally, responsible stakeholders must prioritize the enhancement of mental health services tailored to meet the unique needs of this vulnerable group.

Adolescents with any abuse history had a more likely chance of attempting suicide than adolescents without any physical, sexual, or psychological abuse. This is in line with other studies [99–101]. This might be due to abuse-related trauma, including PTSD, anxiety, depression, social isolation, and impaired coping mechanisms. All these factors predispose adolescents to commit suicide [102]. To address this issue, policy aimed at preventing abuse and ensuring a safe environment for adolescents is critical to mitigating the problem.

## Limitations

This systematic review and meta-analysis determined the pooled prevalence and identified associated factors of suicidal ideation and suicide attempts among high school adolescents in Ethiopia. Nonetheless, the search strategy may have overlooked unpublished studies, and the review did not incorporate qualitative research, potentially excluding key determinants of suicidal ideation and suicide attempts in adolescents. Although subgroup analyses were performed across various study characteristics, substantial heterogeneity persisted, indicating the presence of additional, unexamined influencing factors. Potential sources of heterogeneity may stem from variations in study characteristics or unaccounted confounding variables. However, due to limitations in the available data, further stratified analyses were not feasible. Future research is recommended to include primary qualitative narrative synthesis focusing on country policy lens, urbanization, and resilience factors on suicidal ideation and attempts among adolescents in Ethiopia.

## Conclusion

This systematic review and meta-analysis highlighted that a significant proportion of high school adolescents in Ethiopia experience suicidal ideation and suicide attempts. The findings indicated that studies conducted after the implementation of the mental health strategic plan (HSTP II) reported higher pooled prevalence rates of suicidal ideation and attempts compared to studies conducted prior to the plan. These results underscore the urgent need to strengthen and expand mental health initiatives to address this growing public health concern. Additionally, the successful completion and implementation of the suicide prevention research project in Ethiopia should be prioritized by policymakers and stakeholders.

Several factors, including gender, academic disappointment, family history, alcohol use, the presence or absence of family or social support, anxiety, and depression, were found to be significantly associated with suicidal ideation and suicide attempts. To mitigate these risks, relevant authorities should focus on early diagnosis and treatment of anxiety and depression among adolescents in schools, fostering strong family connections, addressing academic pressures through counseling services, promoting social support systems, and implementing substance use prevention programs. Particular attention should be directed toward vulnerable groups, such as females, orphans, adolescents with a history of alcohol use, and those with mental health challenges.

## Data Availability

The datasets generated during and/or analyzed during the current study are available from the corresponding author on reasonable request.

## Abbreviations

AOR: Adjusted Odds Ratio
CIDI: Composite International Diagnostic Interview
JBI: Joanna Briggs Institute
HSTP: Health Sector Transformation Plan
NOS: Newcastle Ottawa Scale
PRISMA: Preferred Reporting Items for Systematic review and Meta-Analysis
PTSD: Post Traumatic Stress Disorder
WHO: World Health Organization
